# Bromodomain and extraterminal (BET) proteins: biological functions, diseases and targeted therapy

**DOI:** 10.1038/s41392-023-01647-6

**Published:** 2023-11-06

**Authors:** Zhi-Qiang Wang, Zhao-Cong Zhang, Yu-Yang Wu, Ya-Nan Pi, Sheng-Han Lou, Tian-Bo Liu, Ge Lou, Chang Yang

**Affiliations:** 1 Department of Gynecology Oncology, Harbin Medical University Cancer Hospital, Harbin, 150086 China; 2 School of Optometry and Ophthalmology and Eye Hospital, Wenzhou Medical University, Wenzhou, China; 3 Department of Colorectal Surgery, Harbin Medical University Cancer Hospital, Harbin, China

**Keywords:** Drug development, Drug development

## Abstract

BET proteins, which influence gene expression and contribute to the development of cancer, are epigenetic interpreters. Thus, BET inhibitors represent a novel form of epigenetic anticancer treatment. Although preliminary clinical trials have shown the anticancer potential of BET inhibitors, it appears that these drugs have limited effectiveness when used alone. Therefore, given the limited monotherapeutic activity of BET inhibitors, their use in combination with other drugs warrants attention, including the meaningful variations in pharmacodynamic activity among chosen drug combinations. In this paper, we review the function of BET proteins, the preclinical justification for BET protein targeting in cancer, recent advances in small-molecule BET inhibitors, and preliminary clinical trial findings. We elucidate BET inhibitor resistance mechanisms, shed light on the associated adverse events, investigate the potential of combining these inhibitors with diverse therapeutic agents, present a comprehensive compilation of synergistic treatments involving BET inhibitors, and provide an outlook on their future prospects as potent antitumor agents. We conclude by suggesting that combining BET inhibitors with other anticancer drugs and innovative next-generation agents holds great potential for advancing the effective targeting of BET proteins as a promising anticancer strategy.

## Introduction

**Fig. 1:**
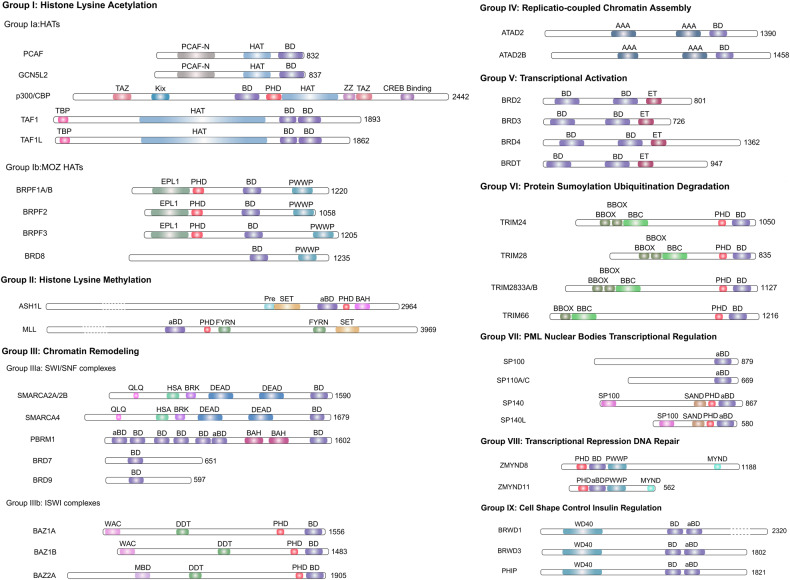
Structural and functional classification of human bromodomain proteins. The figure depicts the functional roles of the nine subclasses of human bromodomain proteins and provides a brief overview of the constituent proteins of each class and their structures. Modified from Zaware^368^

**Fig. 2:**
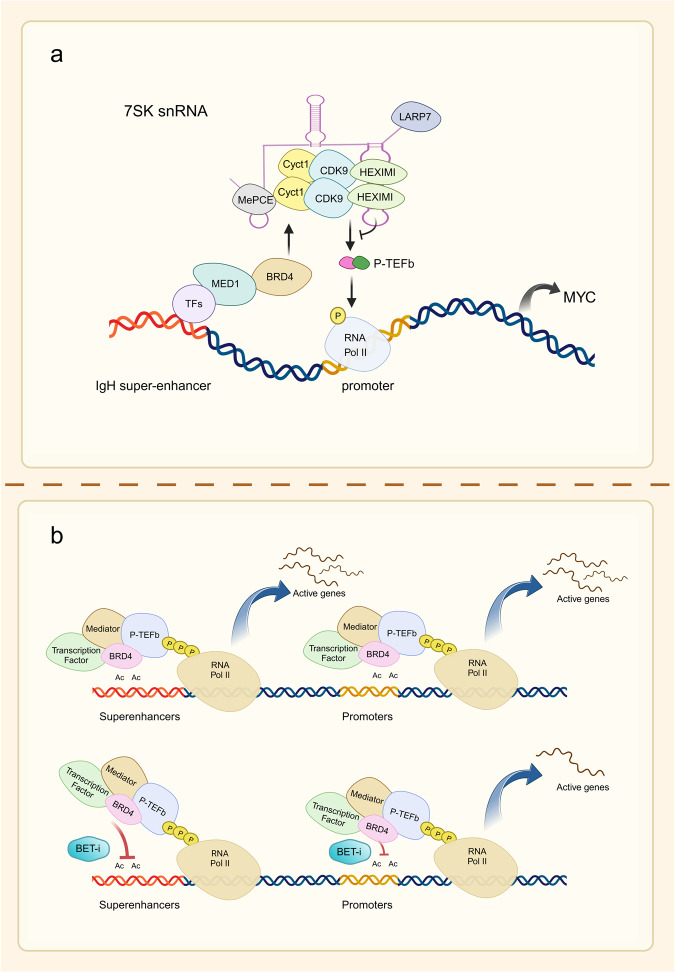
Basis and mechanism of BET inhibitor action. **a** The mechanism by which BRD4 promotes transcriptional elongation. BRD4 can regulate gene transcription in the nucleus through the recruitment of transcription factors. P-TEFb is a complex of cyclin T1 and CDK9 that binds to BRD4 and promotes serine phosphorylation of the C-terminal domain of RNA pol II, driving the transcription process. During gene transcription, BRD4 binds to P-TEFb, displacing the protein HEXIM1/7SK small nuclear ribonucleoprotein, which represses its activity, and converts it to an active elongated state. In the case of the transcription factor c-MYC, for example, BRD4 activates transcriptional initial response genes and is involved in cell cycle, cell proliferation, and apoptosis processes. **b** Schematic representation of the mechanism of action of BET inhibitors. The BET protein recognizes the acetylated lysine on BRD4 and acts as a backbone to recruit mediators, transcription factors, and P-TEFb to the promoter or enhancer, phosphorylating RNA Pol II at the target gene promoter or enhancer to drive the transcription process. BET inhibitors reduce BRD4 levels at the promoter and enhancer at a genome-wide level in tumor cells, and this inhibition is more pronounced at the super-enhancer compared with at the normal enhancer, targeting oncogenes more efficiently and specifically

BET inhibitors are novel targeted medications in the research phase that regulate epigenetic modifications in the therapy of malignant tumors.^1^ Numerous investigations have demonstrated in the past few years that epigenetic modifications perform an essential function in tumor formation. Inhibitors targeting these epigenetic modification-related proteins can suppress overexpressed oncogenes, thus acting as potential antitumor agents. BET is a family of proteins with bromodomains (BRDs), which are distinguished by the presence of conserved BD1 and BD2 sequences at their N-terminals, as well as an extraterminal (ET) structure at the C-terminal (Fig. 1). They identify acetylated residues on histone H3 and H4 and have a stronger affinity when there are multiple acetylated fragments of 1–5 amino acids.^2^ BRD2, BRD3, BRD4, and BRDT are the four members of the BET proteins. Among them, the most powerful and well-researched BET protein is BRD4, known as the “reader” of lysine acetylation. It acts as a regulator of transcription factors that specifically bind to acetylated histone tails, recruit tumor-associated target genes, and act on the promoter and/or enhancer regions.^3^ Throughout the genome, the initiation of transcription for numerous genes is blocked by the interaction between ribonucleic acid polymerase II (RNA Pol II) and the site where transcription begins. RNA Pol II is released when BRD4 binds to an acetylated histone located at the site where transcription begins. The principle is that upon binding to active acetylated chromatin, it can replace the protein HEXIM1/7SK (small nuclear ribonucleoproteins), which inhibits its activity by binding with the positive transcriptional elongation factor (P-TEFb) through the BD2 sequence. As a result, RNA Pol II undergoes serine phosphorylation, which transforms it into an active, elongated state.^4,5^ A complex including BRD4 and various proteins acts as an intermediate and interacts with RNA Pol II, linking the enhancer to Pol II activation (Fig. 2a). Therefore, The BRD4 protein serves as a pivotal transcriptional regulator and is implicated in the control of gene expression for several super-enhancer-associated genes, such as the prominent oncogene c-MYC.^6^ This suggests that the manipulation of BET family proteins might have significant potential as a viable strategy for cancer treatment.

In this paper, we review the biological properties and structural features associated with BET proteins, as well as the advancements achieved with the investigation of BET inhibitors. The initial topic of discussion pertains to the preclinical foundation for the therapeutic targeting of BET proteins in the context of cancer. Subsequently, a review is conducted on the many small-molecule inhibitors of BET proteins that have been investigated thus far. The review places particular emphasis on inhibitors that selectively target the BD1 and BD2 domains, as well as proteolysis targeting chimera (PROTAC) degraders. We review the early findings from clinical studies with BET inhibitors used alone as well as in tandem with various other medicines and discuss the potential for these drugs to serve as antineoplastics in the future. We also highlight the emerging issues of therapeutic resistance and adverse drug events and offer reasonable solutions to these problems. We offer an extensive review of studies involving this novel protein inhibitor in conjunction with a wide variety of anti-cancer therapies. This article underscores the potential of targeting BET proteins for addressing various diseases and enhancing human health.

## Bromodomains: the epigenetic modification readers

### Bromodomain development and discovery

Initially discovered in the Brahma gene of Drosophila melanogaster in 1992, bromodomains are 110-amino-acid protein modules and possess the ability to identify acetylated lysine residues present in histones and other proteins.^3,7,8^ Lysine acetylation (Kac) is a significant and prevalent post-transcriptional modification of proteins that plays a crucial role in the control of gene transcription, metabolic processes, and cell signaling.^9–12^ As scaffolding for the construction of macromolecules that modify the accessibility of chromatin for transcription factors and permit recruiting and the activation of RNA polymerases, BRDs influence transcriptional control by detecting acetylation marks on histones.^2,13^

Subsequent investigations have shown the significant involvement of BET proteins, particularly BRD4, in the pathogenesis and advancement of cancer. This discovery generated interest in utilizing BET proteins for cancer therapy.^14–19^ In a 2008 patent (PCT/JP2008/073864), BET inhibitors were initially described as agents that exhibit antitumor activity by diminishing the interaction between BD-containing proteins and acetylated histones. Two seminal articles revealed the cancer-fighting and inflammation-reducing effects of BET inhibitors in 2010.^1,20^ The subsequent identification of an increasing number of BET inhibitors, which showed substantial preclinical anticancer efficacy in an extensive number of solid and hematological tumors, led to the initiation of clinical trials for a variety of cancer therapies. The purpose of these studies was to evaluate the feasibility of combining BET inhibitors with other therapies in terms of effectiveness, security, and potential. They have also shown efficacy in treating other conditions, including inflammatory and cardiovascular diseases. Over the years, research on BET proteins, their biological functions, diseases, and targeted therapies has increased exponentially, providing a deeper understanding of their role and therapeutic potential. Ongoing research persists in investigating novel applications and combination therapies involving BET inhibitors, providing promising prospects for enhanced treatments in the future.

### Structure of bromodomains

The human genome has a total of 61 BRDs, which are found throughout 46 distinct proteins.^3^ The proteins under consideration are characterized by their globular folding pattern, whereby they exhibit four α-helical tufts referred to as αZ, αA, αB, and αC.^2^ Two interhelical loops, ZA and BC, are present, which together provide a joining region for acetyl-lysine at one extremity of the helical bundle.^2,21^ While the ZA loop connects the B and Z helices, the BC loop binds A and C. A highly conserved asparagine residue in the BC loop creates a hydrogen bond that links the amide nitrogen and the acetyl-lysine carbonyl oxygen.^2,22^ The conserved tyrosine and asparagine residues are of paramount importance in the recognition of lysine acetylation. BRDs consist of a very conserved structural domain at the N-terminus accompanied by a divergent structural domain at the C-terminus, and this modular structure makes bromodomains the functional unit of protein interaction.^23^ Asparagine is present in 48 of the 61 BRDs in the human genome, whereas aspartic acid, tyrosine, and threonine are present in the other 13 BRDs. Those BRDs that include asparagine are considered “typical”, whereas the others are “atypical”.^24,25^

The BET family of proteins contains the BRD proteins BRD2, BRD3, BRD4, and BRDT, which form subfamily V of the BRD family (Fig. 1). BET differs from other BRDs by having two highly conserved tandem bromodomains (BD1 and BD2) at the N-terminal end and an extraterminal structural domain located at the C-terminal end. BRD4 and BRDT possess an extra C-terminal domain (CTD).^20,26,27^ The N-terminal structural domains, namely BD1 and BD2, serve as binding sites for acetylated lysine. The ET domain facilitates the recruitment of transcriptional cofactors and enhances the process of transcription. Additionally, the C-terminal domain plays a role in the recruitment of P-TEFb.^2,28^

### Functions of bromodomains

Bromodomain proteins play crucial roles in several physiological processes and participate in interactions between proteins as well as between proteins and nucleic acids.^2^ Based on their cellular roles, BRDs may be categorized into nine primary groups (Fig. 1).^3,29^ These are, I. Histone lysine acetylation^23,30–32^; II. Histone lysine methylation^23^; III. Chromatin remodeling^33–35^; IV. Replication-coupled chromatin assembly^36,37^; V. Transcriptional activation^29,38,39^; VI. Protein sumoylation, ubiquitination, degradation^21,40,41^; VII. Promyelocytic leukemia (PML) nuclear bodies, transcriptional regulation^39,42,43^; VIII. Transcriptional repression, DNA repair^44,45^; IX. Cell shape control, insulin regulation.^46^ Among bromodomain proteins, the BET family has received the greatest attention and research, with BRD4 being the most characterized member.

#### BRD2

BRD2, formerly named RING3, is the earliest mammalian BET protein that has been functionally acknowledged.^47^ It operates as an atypical protein kinase, mostly localized inside the nucleus.^48,49^ Its primary role involves the activation of E2F1 and E2F2 proteins, hence facilitating the production of proteins essential for the G1/S phase transition.^47,48^ The protein in question has an affinity for the H3 and H4 domains of histones that have undergone acetylation. Its primary function involves the modulation of transcriptional activity via the recruitment of various transcription factors, co-activators, and repressors.^50^ During chromatin remodeling, BRD2 recruits many proteins, such as histone deacetylases.^51,52^ Research has shown that BRD2 is at its peak during the development and closure of the neural tube, and it is essential for both neurogenesis and embryogenesis.^53,54^ Researchers have noted in recent years that BRD2 is associated with improvements in insulin signaling as well as metabolic diseases.^55^

#### BRD3

The gene BRD3, alternatively called ORFX or FSHRG2, has the ability to interact with acetylated lysine residues found in the GATA1 transcription factor.^56^ This interaction plays a crucial role in regulating the expression of genes specific to the red lineage and megakaryocytes.^56,57^ It has been shown to be increased in activated lymphocytes, which points to the possibility of it playing a role in adaptive immunity.^58^ People who suffer with osteoarthritis and rheumatoid arthritis have also had this protein found in the synovial tissue, particularly in macrophages.^59,60^ This observation strongly indicates a potential significance of this molecule in the development of immune disorders. BRD3 contributes to the G1/S transition of dividing cells by controlling the transcription of the cell cycle protein D1.^49^

#### BRD4

BRD4, previously identified as MCAP, FSGRG4, or Hunk1, is extensively found in almost all types of tissues, mainly in the nucleus, and shares 80% of its amino acid sequence with BRD2.^49,61,62^ BRD4 is an important cell cycle regulatory protein that binds to chromosomes during mitosis and performs a crucial function in the regulation of the cell cycle, embryonic development, and maintaining genome stability.^16,19,62–64^ BRD4 plays a role in the activation of the P-TEFb complex by phosphorylating CDK9, which is the complex’s active motif. The phosphorylation of RNA Pol II is a consequence of this activation, thus regulating both transcription initiation and elongation.^65–67^ BRD4 may operate as a transcriptional repressor in addition to being an activator of transcription, according to recent research.^66^ The BRD4 protein has kinase activity, and its extraterminal domain has the capability to interact with SWI-SNF and CHD2 proteins, two proteins that are involved in ATP-dependent chromatin remodeling.^28,68^ The role of BRD4 in the appropriate activation of that nonhomologous end-joined pathway is of significant importance. Its influence on the DNA damage repair system relies on its capacity to establish a platform connecting components of histone modifications and DNA repair mechanisms.^69,70^ BRD4 significantly contributes to telomere maintenance through the recruitment of telomerase and telomerase-associated complexes to the telomeric ends.^71,72^ This recruitment facilitates the extension of telomeres and stabilizes them through the accumulation of acetylated chromatin at the telomeric termini.^71,72^

#### BRDT

BRDt is a bromodomain protein that exhibits specificity for the testis, being expressed only within this particular organ.^73,74^ Studies conducted on animals have demonstrated that the absence of the BRDt protein leads to infertility, low sperm count, and abnormalities in sperm morphology.^75^ Cell cycle protein A1 is a crucial regulating gene in the male germline that is required for sperm cells to enter the first meiotic division, and the expression of cell cycle protein A1 is initiated and regulated by BRDt.^76,77^

## Mechanism and targets of action of BET inhibitors in tumors

BRD proteins exert a significant influence on regulating many critical oncogenes, including MYC, inside cancer cells. The specific targeting and modulation of key pathways in tumor progression, including the JAK/STAT and NF-κB signaling pathways, could be achieved by the inhibition of BRD proteins. BET inhibitors work by displacing BRD4 out of super-enhancer regions, which has a significant impact on vital pathways in cancerous cells. Super-enhancers, as a large set of enhancer clusters, can significantly regulate gene expression, occupying areas of up to 50 kb, far exceeding the coverage of several hundred bases of normal enhancers.^78,79^ In cancer, super-enhancers are mainly found in oncogenes and genes associated with tumor progression.^78,79^

### Numerous genes’ expressions are regulated by BET inhibitors

Malignancies arise from genetic mutations and are associated with abnormal epigenetic regulation of chromatin. Histone modifications are important for the expression of genes. Abnormal histone acetylation can affect cell differentiation and apoptosis, which can result in the increase of transcription for pro-oncogenic growing genes. BRD4 plays a regulatory role in transcriptional elongation, mainly by binding to P-TEFb, which promotes RNA Pol II to become phosphorylated in order to aid in the transcriptional elongation of genes. The key regulatory mechanism of BRD4 is the promotion of *c-MYC* transcription.^4,5,80,81^ The analysis of genetic mutations in primary, metastatic, or recurrent ovarian cancers has shown that c-MYC gains are potential targets of BET inhibitors.^82,83^ Additionally, BRD4 plays a significant role in several cellular processes, such as cell differentiation, signal transmission, control of proto-oncogenes, modulation of the cellular cycle, and other pathophysiological processes through non-histone acetylation modifications. This suggests that the BD of BRD4 selectively inhibits the transcription of genes relevant to malignancy through binding acetylated histones as well as interacting with acetylated lysine residues from various proteins (Fig. 2b).^84^

Furthermore, BET inhibitors have other regulatory targets than c-MYC. Previous studies have reported numerous BET inhibitor-regulated genes in different cancers.^81,85,86^ BET inhibitors primarily function in hematologic malignancies by suppressing the transcriptional expression of BCL2 and c-MYC, two proto-oncogenes.^4,80^

BET inhibitors mainly regulate the signaling pathway known as NF-κB in non-small-cell lung cancer by targeting BRD4.^87^ By preventing BRD4 and the androgen receptor from directly interacting, BET inhibitors in prostate cancer slow the development of tumors and lower the expression of genes that are targets of androgen.^88^ Research on breast cancer indicates that BET inhibitors primarily prevent BRD4 from attaching to the BR-α promoter.^89^ In ovarian cancer studies, BET inhibitors have shown different mechanisms of action depending on the subtype of ovarian cancer and the genetic alterations present in the tumors. Utilizing cytology and ovarian cancer xenograft (PDX) models, Zhang et al. demonstrated that for sensitive ovarian cancer cells, BET inhibitors specifically suppressed FoxM1 and its subsequent targets by cell cycle block, while c-MYC expression was just temporarily downregulated and then returned to baseline levels. In patients with homologous recombination repair defects, Yang et al. observed that BET inhibitors directly act on *BRCA1* and *RAD51* to reduce the efficiency of homologous recombination, leading to reduced PARP-induced repair of DNA-damaged tumor cells, which results in apoptosis.^90^ In addition, studies have shown that BRD4 can interact with CtIP to reduce CtIP expression, and independent of BRCA1/2, RAS, or BRAF mutations, multiple tumor cell lines are more sensitive to PARP inhibitors.^91^ Moreover, Villar-Prados et al. demonstrated, through an advanced drug target screening platform (TPT) and an experimental approach, that *NOTCH3* is significantly upregulated in ovarian carcinoma, which was shown to be closely linked to cell proliferation, chemoresistance, and apoptosis. BET inhibitors have the capability to selectively modulate the expression of NOTCH3 by targeting BRD4 and affect its downstream effector genes.^92^ Liu et al. showed that BET inhibitors can upregulate the pro-apoptotic factor BIM (BCL2L11) (of the BCL2 family), cause tumor cells to undergo apoptosis via the mitochondrial apoptotic pathway, and inhibit tumor cell invasion and migration through the STAT3 pathway by downregulating its phosphorylation. They also found that inhibition of STAT3 affects the immune microenvironment by increasing CD3+ and CD8+ lymphocytes and promoting tumor cell immunogenic death.^93^ Recent research indicates that tumor-associated macrophages are crucial to the development of ovarian cancer, with high expression of BRD4 upregulating CSF1 to promote macrophage proliferation and differentiation. BET inhibitors significantly inhibit CSF1 production by tumor cells, which could affect TAM-mediated cancer progression.^94^

### JAK/STAT pathway targeted by BET inhibitors

JAK/STAT signaling is a crucial process in cell proliferation and differentiation, facilitated by extracellular cytokine-activated receptors. It is thought that disruption of this pathway plays a significant role in the development of malignant tumors.^95^ Through forming a heterodimer with the interleukin-7 receptor (IL7R), CRLF2 promotes proliferating and inhibits apoptosis in B-cell ALL cells through signaling through JAK2, JAK1, and STAT5.^96^ JQ1, a BET inhibitor, downregulates the transcription of IL7R, displaces BRD4 out of the IL7R promoter, and also suppresses the activation of JAK2 as well as STAT5.^96^ OTX015, a BET inhibitor, was found to negatively regulate intermediaries of the JAK/STAT signaling pathway in in vitro and in vivo experimental studies of mature B-cell lymphomas.^97^ Consequently, BET inhibitors present a promising therapeutic approach for disorders that are dependent on JAK/STAT signaling.

### BET inhibitors target the NF-κB pathway

The dysregulation of NF-κB activity plays a significant role in the development and advancement of malignancy.^98^ The phosphorylation of the IκB kinase (IKK) complex constitutes a crucial step in the activation of the typical NF-κB pathway.^99,100^ Research into ABC DLBCL has shown that BET inhibitors reduce IKK activity; therefore, these inhibitors hinder the subsequent NF-κB-driven transcription processes and induce ABC DLBCL cell death.^101^ JQ1, a BET inhibitor, prevents BRD4 from binding to Ac-Lys310, hence suppressing NF-κB activation and NF-κB-related transcription.^87,102^ A study on melanoma found that BRD4 regulates SPP1 expression via NFKB2 and BET inhibitors stop the growth of carcinoma via the noncanonical NF-κB/SPP1 pathway.^103^ These results imply that BET inhibitors might be a useful therapeutic strategy for the management of malignancies caused by NF-κB.

## Small-molecule BET protein inhibitors

BRD recruits several additional proteins to active genes that induce transcription by recognizing acetylated lysine residues in histones and transcribed proteins.^23,104^ The main way that BET proteins regulate transcription is by interacting with acetylated chromatin.^105^ Because of their significant significance in the pathophysiology of several diseases, BET proteins, a unique category of BD proteins, have the potential to be effective therapeutic targets. Numerous small-molecule compounds were formulated with the specific purpose of targeting BET proteins, presenting potential treatments for cancer, inflammation, cardiovascular diseases, and autoimmune disorders. These compounds hold promise for diverse cancer types, especially when employed alongside other small-molecule inhibitors and epigenetic modulators, yielding promising outcomes in certain cases. Nonetheless, the obstacles of pharmaceutical resistance and side effects persist, impeding the progress of BET inhibitors in clinical applications.^106–111^ The pursuit of enhancing medication effectiveness and minimizing drug-related adverse effects has led to the development of novel selective molecules. This section presents a detailed examination of several classes of BET inhibitors, elucidating their prospective applications and potential for novel drug development.

### Non-selective BET inhibitors

A 2008 patent (PCT/JP2008/073864) first detailed the antitumor activity of BET inhibitors, which involves reducing the interaction of BRD proteins with acetylated histones. Two landmark investigations carried out in 2010 demonstrated that BET inhibitors have anticancer and anti-inflammatory properties.^1,20^ Subsequently, more new BET inhibitors have been discovered, exhibiting significant preclinical anticancer efficacy in many types of solid and hematological tumors.^23,112,113^

JQ1, regarded as a pan-BET inhibitor with similar inhibiting effects against BD1 as well as BD2, was among the earliest BET inhibitors to become available.^1^ Differential scanning fluorometry revealed that JQ1 had the highest binding affinity for BRD4 among 41 human BD-containing proteins.^1^ By interacting with BRD4’s acetylation lysine binding region and removing BRD4 from nuclear chromatin, it induces differentiation and suppresses tumor development in BRD4-dependent carcinomas.^1^ In animal models, JQ1 has also shown promising pharmacodynamic and antitumor.^1,114^ Considering that JQ-1 has a brief in vivo half-life and hence modest translational prospective,^1^ numerous BET inhibitors, including OTX015, i-BET762, and NHWD-870, were recently developed and clinically investigated to evaluate their safety and effectiveness among individuals with hematologic malignancies or solid tumors (Table 1).

**Table 1:** Clinical trials with BET inhibitors in monotherapy (source: https://clinicaltrials.gov)

Phase	Drug	Conditions	Trial#	Status
I	ABBV-075	BC, NSCLC, AML, MM, PC, SCLC, NHL	NCT02391480	Completed
I	ABBV-075	Myelofibrosis	NCT04480086	Active, not recruiting
I	ABBV-744	AML	NCT03360006	Completed
I	ABBV-744	Myelofibrosis	NCT04454658	Recruiting
I	BAY1238097	Neoplasms	NCT02369029	Terminated
I	BMS-986158	Pediatric Cancer	NCT03936465	Recruiting
I	BMS-986378	Pediatric Cancer	NCT03936465	Recruiting
I	BMS-986378	Astrocytoma, Glioblastoma	NCT04047303	Active, not recruiting
I	BMS-986378	NHL	NCT03220347	Active, not recruiting
I	CPI-0610	Multiple Myeloma	NCT02157636	Completed
II	CPI-0610	Peripheral Nerve Tumors	NCT02986919	Withdrawn
I	CPI-0610	Progressive Lymphoma	NCT01949883	Completed
II	CPI-0610	Acute Leukemia, Myelofibrosis	NCT02158858	Recruiting
I	CPI-0610	Advanced Malignancies	NCT05391022	Recruiting
II	I-BET762	Relapsed, Refractory Hematologic Malignancies (AML, NHL, MM)	NCT01943851	Completed
I	I-BET762	NMC and Other Cancer	NCT01587703	Completed
I	I-BET762	NMC	NCT03702036	No longer available
I	OTX015	Hematologic Malignancies	NCT01713582	Completed
II	OTX015	Glioblastoma Multiforme	NCT02296476	Terminated
II	OTX015	NMC, TNBC, NSCLC, CRPC	NCT02698176	Terminated
I	OTX015	PDAC	NCT02259114	Completed
I	OTX015	AML, DLBCL	NCT02698189	Completed
I/II	PLX51107	Steroid Refractory GVHD	NCT04910152	Recruiting
I	PLX51107	AML, Solid tumors, MS, NHL	NCT02683395	Terminated
I	RO6870810	AML, MS	NCT02308761	Completed
I	RO6870810	Multiple Myeloma	NCT03068351	Completed
I	RO6870810	Solid Tumors, Advanced Solid Tumors	NCT01987362	Completed
I/II	Rvx000222	Fabry Disease	NCT03228940	Unknown
II	Rvx000222	Diabetes	NCT01728467	Completed
I/II	Rvx000222	Chronic Kidney Failure	NCT03160430	Unknown
II	Rvx000222	Atherosclerosis, Coronary Artery Disease	NCT01058018	Completed
III	Rvx000222	Type 2 Diabetes Mellitus, Coronary Artery Disease	NCT02586155	Completed
I/II	Rvx000222	Dyslipidemia, Atherosclerosis, Cardiovascular Disease	NCT00768274	Completed
II	Rvx000222	Dyslipidemia, Coronary Artery Disease	NCT01423188	Completed
II	Rvx000222	Coronary Artery Disease	NCT01067820	Completed
I	SYHA1801	Advanced Solid Tumors	NCT04309968	Recruiting
I	TQB3617	Advanced Malignant Tumors	NCT05110807	Recruiting
I	ZEN-3694	Metastatic CRPC	NCT02705469	Completed
II	ZEN-3694	Squamous Cell Lung Cancer	NCT05607108	Recruiting
I/Ib	ZEN-3694	Solid Tumor	NCT04840589	Recruiting
I	EP31670	Solid Tumor NUT Carcinoma	NCT05488548	Recruiting
I	MK-8628	NMC Solid Tumor	NCT02259114	Completed
I	MK-8628	AML	NCT02698189	Terminated
I	MK-8628	NMC Solid Tumors	NCT02698176	Terminated
I	MK-8628	AML	NCT02698189	Terminated
II	MK-8628	Glioblastoma Multiforme	NCT02296476	Terminated
Ib	CC-95775	Lymphoma, Non-Hodgkin	NCT04089527	Completed
I/Ib	FT-1101	AML/MDS/NHL	NCT02543879	Completed
I/II	GSK525762	Hematological Malignancies	NCT01943851	Completed
I/II	GSK525762	NMC	NCT01587703	Completed
I/II	INCB054329	Solid Tumors, Hematologic malignancy	NCT02431260	Eliminated by the sponsor due to PK variability

*AML* acute myeloid leukemia, *BC* breast cancer, *CRPC* castration-resistant prostate cancer, *GBM* glioblastoma multiforme, *GVHD* graft versus host disease, *HGBL* high-grade B-cell Lymphoma, *MM* Multiple Myeloma, *MPNST* malignant peripheral nerve sheath tumor, *MS* myelodysplastic syndrome, *NSCLC* non-small cell lung cancer, *NHL* non-hodgkin’s lymphoma, *NMC* NUT midline carcinoma, *OC* ovarian cancer, *PDAC* pancreatic ductal adenocarcinoma, *SCLC* small cell lung cancer, *TNBC* triple-negative breast cancer

OTX015 is a small-molecule inhibitor of BRD2, BRD3, and BRD4 with a structure comparable to that of JQ1, and an important advancement is its oral administration.^97,115,116^ In a 2016 clinical trial, the effects of OTX015 on AML and ALL were examined.^117^ Exposure to OTX015 caused a potent decrease in BRD2, BRD4, as well as c-MYC, along with an increase in HEXIM1 protein levels, whereas BRD3 expression remained unchanged.^118^ These changes suggest that OTX015 may lead to cell cycle arrest, apoptosis, and growth suppression.^119^ Additionally, research conducted in vitro has shown that OTX015 has promising synergistic effects with several anticancer drugs, particularly mTOR and BTK inhibitors.^97^

I-BET is a synthetic that “mimicks” acetylated histones to disrupt chromatin complexes that are essential to the transcription of important genes associated with inflammation in activated macrophages.^20^ Previous studies have demonstrated that it provides a safeguard against lipopolysaccharide-induced endotoxic shock and bacterial-induced sepsis.^20^ I-BET762 is an orally bioavailable BET inhibitor with pan-affinity for BET proteins, primarily targeting BRD2, BRD3, and BRD4.^20,120^ Studies have demonstrated that I-BET762 acts mainly through the downregulation of MYC and IRF4, as well as the upregulation of HEXIM1.^121^

NHWD-870, a recently developed BET inhibitor, demonstrates notable efficacy in suppressing the proliferation of multiple cancers by downregulating the expression of macrophage CSF1 in tumor cells. In comparison to three prominent BET inhibitors currently in clinical development, namely BMS-986158, OTX015, and GSK525762, NHWD-870 exhibits greater potency, as evidenced by a cytometric assay.^94^ Currently, NHWD-870 is undergoing Phase I clinical trials for the treatment of various kinds of cancer (CXHL200250). Through controlling SPINK6, NHWD-870 has been shown in recent studies to significantly diminish melanoma invasion and metastasis both in vivo and in vitro,^122^ and NHWD-870 in combination with cytarabine demonstrated synergistic effects against melanoma both in vivo and in vitro.^123^ NHWD-870 has also shown excellent therapeutic potential in the treatment of osteosarcoma.^124^ NHWD870 exhibited a favorable contraceptive effect in in vivo animal experiments, and upon discontinuation of the drug, fertility can be restored to its baseline. This suggests a potential future role for NHWD870 as a male contraceptive. Nonetheless, its safety and efficacy require further investigation in preclinical studies.^125^

Notably, the overall remission rate (complete/partial remission or stable disease status) for pan-BET inhibitors is less than 30% in most clinical trials based on current data.^117,126–128^ This low response rate might have something to do with the intermittent dosing schedule, whose purpose was originally designed to reduce targeted side effects such as thrombocytopenia.^117,126,127^ The primary challenge encountered in the development of pan-BET inhibitors is balancing the enhancement of drug efficacy with the reduction of side effects. Consequently, an important direction of BET inhibitor research is advancing the development of more potent or selective BET inhibitors.

### Selective BD1 inhibitors

While the sequence similarity of BD1 and BD2 exceeds 75%, the similarity of their structural domains is merely 38%.^129^ The lack of specificity of pan-BET inhibitors towards BRD proteins and BD1 as well as BD2 has resulted in the occurrence of several undesirable effects. Consequently, enhancing selectivity by inhibiting either BD1 or BD2 exclusively may offer a potential solution to boost specificity, avoid adverse effects, and improve the clinical efficacy of BET inhibitors.^23^

MS436, the earliest identified small nanomolar BD1 inhibitor, has a selectivity that is ten times higher towards BRD4-BD1 compared to BRD4-BD2.^130^ The selectivity of MS436 towards BD1 is associated with the hydrogen bonding interactions between MS436 and certain amino acid residues, including Pro82, Gln85, Lys91, and Asn140.^130^ In mouse macrophages, MS436 effectively inhibited the transcriptional activity of BRD4 in the NF-κB-driven generation of the pro-inflammatory cytokines IL6 and nitric oxide.^130^ The subsequent inhibitor of this series, MS402, is nine-times more specific for BD1 than BD2.^131^ It effectively inhibits and mitigates colitis generated by T-cell metastasis in mice by impeding the excessive proliferation of Th17 cells.^131^

Olinone has a 100-fold higher BD1 selectivity than BD2, and Olinone promotes mouse oligodendrocyte progenitor differentiation, whereas JQ1 inhibits oligodendrocyte progenitor differentiation, suggesting that selective regulation of individual BDs enhances cellular regenerative activity in aging and neurodegeneration.^132^

GSK789, being a highly selective BET-BD1 inhibitor, exhibits selectivity for BD1 that is approximately 1000 times better than that of BD2. Comparable to the antiproliferative activities of pan-BET inhibitors, it maintains robust anti-inflammatory and immunomodulatory activity in vitro.^133^

### Selective BD2 inhibitors

RVX-208 was the earliest known BD2-selective BET inhibitor to induce a more specific expression profile compared with the gene expression altered in response to JQ1.^134^ The limited range of genes impacted by RVX-208 diminishes its efficacy as an antitumor medication, but because of its anti-inflammatory effects, it is well tolerated as a treatment for cardiovascular disease and type 2 diabetes.^135–138^

ABBV-744 exhibits a remarkable degree of selectivity towards BD2 in BRD2, BRD3, and BRD4 and has an affinity for BD2 that is hundreds of times higher than that for BD1.^139,140^ In contrast to RVX-208, which has limited efficacy as an anticancer agent, treatment with ABBV-744 produces potent antiproliferative effects in AML and prostate cancer.^139–141^

SJ432 is a novel tetrahydroquinoline-like BD2 selective inhibitor that exhibits higher effectiveness than JQ1 in neuroblastoma models in vivo through sustained inhibition of expression of the driver oncoprotein c-MYC.^142^ This suggests that BD2-selective molecules may retain efficacy while mitigating several kinds of undesirable toxicity linked to excessive dosages of BET inhibitors, providing a strong rationale for the development of BD2-selective BET inhibitors in cancer therapy.

BY27 is a compound that selectively inhibits BD2 and demonstrates promising anticancer properties.^143^ It has been found to effectively inhibit cancer growth in mice while exhibiting lower toxicity compared to pan-BET inhibitors.^143^ The unique structural features of BY27 make it a promising candidate for the development and synthesizing of future BD2 selective inhibitors.^143^

However, there is ongoing debate surrounding the notion of specifically focusing on BD2 as a target for cancer therapy. Some researchers propose that selectively inhibiting BD1, while excluding BD2, is adequate to elicit the desired antitumor effects of pan-BET inhibitors.^141^ This stands in contrast to various BD2-selective BET inhibitors demonstrating potent anticancer activity, necessitating further investigation to reconcile these disparities. Nonetheless, it remains a fact that BD1 and BD2 may play different functions in the proliferation of diverse tissue types. Nevertheless, all the BD2-selective inhibitors have the ability to mitigate some of the undesired toxicity associated with pan-BET inhibitors.^138,139,141,142,144^ This suggests that the development of BET inhibitors that selectively target either the BD1 or BD2 domains might be a viable approach to improving their safety profile.

### BET degraders: PROTACs

**Fig. 3:**
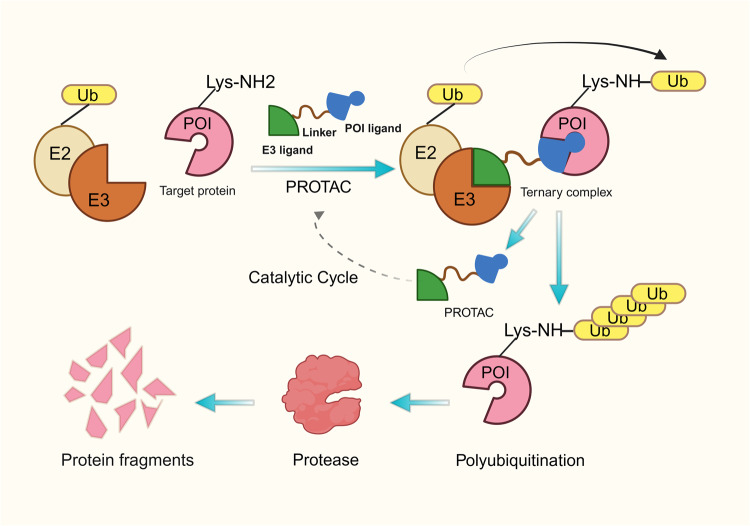
Schematic diagram of proteolysis targeting chimera (PROTAC). The PROTAC molecule consists of a target protein binder, an E3 ligase recruitment element, and a chemical linker. The ligands at either end of PROTAC recognize E3 ubiquitin ligase, and the target protein forms a ternary complex (target protein/PROTAC/E3 ligase). E3 uses the E2 ubiquitin-binding enzyme to transfer ubiquitin to the surface of the target protein, following which the proteasome recognizes the polyubiquitination signal and degrades the target protein, while the PROTAC molecule breaks down and participates in the next degradation cycle

BET inhibitors prevent E3 ligase-mediated identification of the degradation region between BD1 and BD2, resulting in attenuated ubiquitination-dependent degradation of BET proteins, which causes frequent feedback upregulation of BET proteins themselves and leads to back-skipping of c-MYC expression.^97,145^ BET degraders offer a promising strategy for targeting BET proteins to overcome drug resistance by promoting BET protein breakdown to ensure a sustainable block of their function. The strategy is called PROTAC (Fig. 3).^146,147^ Target protein binder, E3 ligase recruitment element, and chemical linker make up the bifunctional small molecule known as PROTAC.^148–151^ The ligands situated on the two termini of PROTAC exhibit recognition capabilities towards the E3 ubiquitin ligase and the target protein, respectively, forming a ternary complex.^152,153^ The formation of the ternary complex facilitates the spatial closeness between the two proteins, leading to the polyubiquitination of the target protein, which subsequently undergoes destruction inside the cell via the action of the proteasome.^152,153^ Currently, cell inhibitor of apoptosis protein, cereblon ligand, murine double minute 2 ligand, and VHL ligand are the major E3 ubiquitin ligases used in PROTAC.^154^

dBET1, the first BET degrader reported in 2015, was developed utilizing a thalidomide derivative and JQ1 as an E3 ligase handle, efficiently and selectively degraded multiple BET family members expressed in leukemic cells, and significantly reduced MYC levels.^147^ dBET1 significantly reduces BRD4 within an hour and completely degrades it in two, whereas JQ1 or thalidomide, as a single treatment, had no effect.^147^ As PROTACs completely deplete BRD4 protein levels and subsequent downregulation of MYC, they exhibit higher in vitro action than BET inhibitors.^155–157^ Although JQ1 is also used as a BD recruitment scaffold, the BET degrader MZ1 exhibits selectivity towards BRD4, suggesting that target selectivity might be improved by the introduction of the E3 ubiquitin ligase VHL.^158^ This is due to MZ1’s ternary crystal structure, which is linked to the VHL ligand via polyethylene glycol chains, allowing the development of particular interactions between molecules within the ternary complex, causing BRD4 to degrade selectively.^159^ MYC, CDK4, cyclin D1, and BTK are all significantly downregulated in a mantle cell lymphoma (MCL) cell line when exposed to BET degraders ARV-825 and ARV-771.^155^ ARV-771 has also shown antitumor efficacy in AML and CRPC models.^160,161^

Although PROTACs hold great promise for drug development, off-targeting, cell permeability, and stability are still significant challenges for clinical applications. One potential problem to overcome is the difficulty of synthesizing hybrid molecules, a process that involves optimizing the size and composition of linkers as well as E3 ligase ligands. Additionally, the complete loss of BET proteins may result in potential adverse effects due to their critical role in physiological states. Hence, once these challenges are overcome, PROTACs are expected to become an important antitumor treatment, ushering in a new era of biopharmaceutical innovation.

## Clinical activity of BET inhibitors

A few single or combination BET inhibitors have been formulated for the therapeutic intervention of hematologic malignancies, NUT midline cancers, and various solid tumors (Tables 1 and 2). Most BET inhibitors are only in Phase I or II, with several having shown mixed outcomes in clinical trials. In this section, we examine published results from BET inhibitor clinical trials and delve into the mechanistic underpinnings of select combinations under investigation.

**Table 2:** Clinical trials of BET inhibitor in combination therapy

Phase	Drug	Combination	Molecular target(s)	Diseases	Trial#	Status
I	ABBV-075	Venetoclax	BCL-2 inhibitor	BC, NSCLC, AML, MM, PC, SCLS, NHL	NCT02391480	Completed
I	ABBV-075	Navitoclax/Ruxolitinib	BCL-2 inhibitor/JAK inhibitor	Myelofibrosis	NCT04480086	Active, not recruiting
I	ABBV-744	Navitoclax/Ruxolitinib	BCL-2 inhibitor/JAK inhibitor	Myelofibrosis	NCT04454658	Recruiting
I/II	AZD5153	Selumetinib/Durvalumab	MEK inhibitor/anti-PD-L1 Ab	MPNST, NF1, Sarcoma	NCT05253131	Not recruiting
I	AZD5153	Olaparib	PARP inhibitor	Lymphoma, OC, BC, PDAC, PC	NCT03205176	Completed
I	AZD5153	Acalabrutinib	BTK inhibitor	NHL, DLBCL	NCT03527147	Completed
I/II	AZD5153	Venetoclax	BCL-2 inhibitor	AML	NCT03013998	Recruiting
I/II	BMS-986158	Ruxolitinib/Fedratinib	JAK inhibitor	Blood Cancer (Myelofibrosis)	NCT04817007	Recruiting
I/II	BMS-986158	Nivolumab	JAK inhibitor	Advanced Solid Tumors or Hematologic Malignancies	NCT02419417	Completed
I/II	BMS-986158	CC-92480/Tazemetostat/ Trametinib/Dexamethasone	CELMoD/EZH2 inhibitor/MEK inhibitor/N/A	MM	NCT05372354	Recruiting
I	BMS-986378	Temozolomide/Radiotherapy	N/A/N/A	GBM	NCT04324840	Recruiting
II	BMS-986378	Cisplatin/Etoposide/ Carboplatin/Nivolumab	N/A/Topoisomerase-II inhibitor/N/A/JAK inhibitor	SCLC	NCT03850067	Active, not recruiting
II	CPI-0610	Ruxolitinib	JAK inhibitor	Acute Leukemia, Myelofibrosis	NCT02158858	Recruiting
III	CPI-0610	Ruxolitinib	JAK inhibitor	Myelofibrosis	NCT04603495	Recruiting
I	FT-1101	Azacitidine	DNMT inhibitor	AML/MDS, NHL	NCT02543879	Completed
I	I-BET762	Entinostat	HDAC inhibitor	Solid Tumors and Lymphomas	NCT03925428	Withdrawn
I/II	I-BET762	Etoposide/ Platinum	Topoisomerase-II inhibitor/N/A	NUT Carcinoma	NCT04116359	Withdrawn
II	I-BET762	Trametinib	N/A	Solid Tumors	NCT03266159	Withdrawn
I/II	I-BET762	Fulvestrant	ER antagonists	HR + /HER2- advanced or metastatic breast cancer	NCT02964507	Completed
I	I-BET762	Abiraterone/Enzalutamide/ Prednisone	CYP17A1 inhibitor/AR inhibitor/N/A	CRPC	NCT03150056	Terminated
I	I-BET762	Itraconazole/Rifampicin	N/A	Healthy Female Subjects of Non Child Bearing Potential	NCT02706535	Completed
I	I-BET762	Entinostat	HDAC inhibitor	NHL, PDAC, Lymphoma	NCT03925428	Withdrawn
I/II	I-BET762	Cisplatin/Etoposide	N/A/Topoisomerase-II inhibitor	Refractory NUT Carcinoma	NCT04116359	Withdrawn
I/II	OTX015	Azacitidine	DNMT inhibitor	AML	NCT02303782	Withdrawn
I	PLX51107	Azacitidine	DNMT inhibitor	AML, MS	NCT04022785	Completed
I	RO6870810	Daratumumab	Anti-CD38 mAb	Multiple Myeloma	NCT03068351	Completed
I	RO6870810	Atezolizumab	anti-PD-L1 Ab	OC, TNBC	NCT03292172	Terminated
I	RO6870810	Venetoclax/Rituximab	BCL-2 inhibitor/Anti-CD20 mAb	DLBCL, HGBL	NCT03255096	Completed
II	RVX000222	Rosuvastatin/Atorvastatin	HMG-CoA reductase inhibitor	Dyslipidemia, Coronary Artery Disease	NCT01863225	Terminated
III	RVX000222	Atorvastatin or Rosuvastatin	HMG-CoA reductase inhibitor	Type 2 Diabetes Mellitus, Coronary Artery Disease	NCT02586155	Completed
II	ZEN-3694	Talazoparib	PARP inhibitor	Advanced Malignant Solid Neoplasm, Advanced OC	NCT05327010	Recruiting
I	ZEN-3694	Pembrolizumab/Nab-paclitaxel	anti-PD-1/N/A	Advanced TNBC	NCT05422794	Recruiting
II	ZEN-3694	Enzalutamide/Pembrolizumab	AR antagonists/anti-PD-1 Ab	Metastatic CRPC	NCT04471974	Recruiting
I	ZEN-3694	Binimetinib	MEK inhibitor	Refractory solid tumors with RAS alterations, TNBC	NCT05111561	Suspended
II	ZEN-3694	Enzalutamide	AR antagonists	Metastatic CRPC	NCT04986423	Recruiting
II	ZEN-3694	Talazoparib	PARP inhibitor	TNBC	NCT03901469	Recruiting
I/II	ZEN-3694	Enzalutamide	AR antagonists	Metastatic CRPC	NCT02711956	Completed
I/II	ZEN-3694	Entinostat	HDAC inhibitor	Lymphoma, Pancreatic cancer, NHL, Advanced Solid Neoplasm	NCT05053971	Recruiting
I/II	ZEN-3694	Enzalutamide	AR antagonists	Metastatic CRPC	NCT04145375	Invitation
II	ZEN-3694	Talazoparib	PARP inhibitor	Peritoneal Cancer, OC, Fallopian Tube Cancer	NCT05071937	Recruiting
I	ZEN-3694	Abemaciclib	CDK4/6 inhibitor	NUT Carcinoma, Metastatic Malignant Solid Neoplasm	NCT05372640	Suspended
I/II	ZEN-3694	Cisplatin	N/A	NUT Carcinoma	NCT05019716	Recruiting
I	ZEN-3694	Nivolumab with or without Ipilimumab	anti-PD-1/anti-CTLA-4	OC, Malignant Solid Neoplasm	NCT04840589	Recruiting
I/II	NUV-868	Olaparib/Enzalutamide	PARP inhibitor/AR antagonists	Solid Tumor	NCT05252390	Recruiting

### Clinical trial results of BET inhibitor monotherapy

In 2014, a Phase 1 dose-escalation trial for OTX015 among individuals with advanced acute leukemia was the first to report the clinical activity of a BET inhibitor.^162^ In 46 individuals with CRPC, NMC, and NSCLC, birabresib (OTX015) was the subject of a Phase Ib research to assess its safety, effectiveness, and pharmacokinetics (NCT02259114).^163^ Birabresib 80 mg one day, administered continuously, is the recommended Phase II dosage for individuals with solid tumors. In the future, research on birabresib should take into account intermittent dosage to potentially reduce the toxicity of long-term therapy since the medication has a favorable safety profile and dose-proportional exposure.

A recent Phase I/II study on molibresib monotherapy for relapsed or refractory hematologic malignancies (NCT01943851) involving 111 patients found that molibresib therapy was tolerated, although gastrointestinal and thrombocytopenic toxicities limited its use. The most frequently occurring side effects included diarrhea (50%), nausea (46%), and thrombocytopenia (40%). A total of 13% of the patients had an objective remission rate, with six of them experiencing full remission and seven experiencing partial remission.^164^

In 2019, individuals with relapsed/refractory solid tumors participated in a first-in-human trial involving mivebresib (ABBV-075).^165^ Mivebresib was administered to 14 individuals with solid tumors during dosage escalation, subsequent to an expanded cohort of 49 individuals with prostate cancer. The most frequent side effects linked to mivebresib that occurred as a result of therapy were dysgeusia (49%), thrombocytopenia (48%), fatigue (26%), and nausea (25%). Of the 61 patients evaluable for dose escalation, 26 (43%) had stable disease and 35 (57%) had progressive disease. The median progression-free survival was 1.8 months (95% CI: 1.8–1.9).

A Phase 1/2a open-label study (NCT02419417) evaluated the safety, tolerability, pharmacokinetics, and pharmacodynamics of BMS-986158.^166^ For the purpose of dose escalation, three BMS-986158 dosimeters were used, namely A, B, and C. Dosimeter A was administered for a duration of 5 days, followed by 2 days of rest, with a dosage range of 0.75–4.5 mg. Dosimeter B was administered for a duration of 14 days, followed by 7 days of rest, with a dosage range of 2.0–3.0 mg. Dosimeter C was administered for a duration of 7 days, followed by 14 days of rest, with a dosage range of 2.0–4.5 mg. A total of 83 patients were included in the study, and the most treatment-associated side effects observed were diarrhea (43%), followed by thrombocytopenia (39%), with lower rates of treatment-related adverse events in schedules A (72%) and C (72%) compared with schedule B (100%). Stable disease was seen in 12 individuals (26.1%) after treatment with schedule A, in 3 individuals (37.5%) following treatment with schedule B, and in 9 individuals (31.0%) following treatment with schedule C. The study demonstrated that Plan A dosing had acceptable tolerability, preliminary anticancer efficacy, and dose-proportional pharmacokinetics.

A first-in-human Phase I study of 64 patients with relapsed/refractory lymphoma reported on the safety, pharmacokinetics, pharmacodynamics, and preliminary clinical activity of CPI-0610 (NCT01949883).^167^ Pelabresib (CPI-0610) was found to be a selectivity and potency small-molecule inhibitor of BET proteins at a maximum tolerated dose (MTD) of 225 mg once daily for 14 days with a 7-day break, with a clear pharmacokinetic/pharmacodynamic relationship and a manageable clinical safety profile. Pelabresib was able to inhibit BET target genes in a dose-dependent manner, with inhibition of IL8 and CCR1 mRNA at doses above 120 and 170 mg, respectively.

A Phase 1 study was conducted in patients with NMC, other solid tumors, or DLBCL with MYC dysregulation using a 21-day or 14-day cycle of subcutaneous RO6870810 injections, respectively (NCT01987362).^168^ Fatigue (42%), decreased appetite (35%), and injection site erythema (35%) were the most common treatment-emergent adverse events. This study demonstrated the safety, favorable pharmacokinetics, evidence of target engagement, and preliminary single-agent activity of RO6870810.

The safety and efficacy of ZEN-3694 plus enzalutamide in metastatic CRPC (mCRPC) were evaluated in a Phase Ib/IIa study.^169^ Of the 75 patients enrolled, 30 (40.0%) were resistant to abiraterone, 34 (45.3%) to enzalutamide, and 11 (14.7%) to both. Fourteen patients (18.7%) experienced grade ≥3 toxic reactions, including three patients with thrombocytopenia (4%). The trial findings indicate that the combination of ZEN-3694 and enzalutamide exhibits satisfactory tolerability and promising effectiveness in individuals diagnosed with mCRPC who have developed resistance to inhibitors targeting androgen signaling.

### Results from clinical trials of BET inhibitors in combination with other drugs

In relapsed/refractory AML patients, a Phase 1 research examined the security and effectiveness of the BET inhibitor mivebresib (ABBV-075) as monotherapy (MIV-mono) or in combination with venetoclax (MIV-Ven). Of the 44 treated patients, 19 received MIV-mono and 25 received MIV-Ven. The study reported that the most common treatment-emergent adverse events associated with mivebresib were dysesthesia (74%), decreased appetite (42%), and diarrhea (42%), in the MIV-mono group, and decreased appetite (44%), vomiting (44%), and nausea (40%), in the MIV-Ven group. In the MIV-mono group, one patient achieved complete remission with incomplete recovery of blood counts, and 15 patients experienced resistance to the drug. In the MIV-Ven group, two patients were in complete remission, two were in partial remission, two had no morphological leukemic status, one was aplastic, and twelve were drug-resistant. The research findings indicated that mivebresib exhibited favorable tolerability and demonstrated anti-leukemic efficacy, both when administered as a standalone treatment and when used in conjunction with venetoclax.^127^

Patients with JAKi-naïve myelofibrosis received pelabresib (CPI-0610) with ruxolitinib in a Phase 2 MANIFEST trial (NCT02158858).^170,171^ Eighty-four patients received ≥1 dose of pelabresib and ruxolitinib. The combination of pelabresib and ruxolitinib demonstrated improved splenic and symptomatic responses, as well as improved myelofibrosis outcomes, in both ruxolitinib-naïve and formerly treated individuals. Furthermore, this combination therapy exhibited a favorable safety profile. Currently, the Phase 3 MANIFEST-2 trial is underway to assess the efficacy of pelabresib in combination with ruxolitinib specifically in myelofibrosis individuals who have not responded to previous JAKi therapy (NCT04603495).

The results of a Phase 1b dose-escalation study of a novel subcutaneous BET inhibitor, RO6870810, in combination with the BCL2 inhibitors venetoclax and rituximab in relapsed/refractory DLBCL were recently reported (NCT03255096).^172^ Dose-limiting toxicities included grade 3 febrile neutropenia, grade 4 diarrhea, and hypomagnesemia with the RO6870810 + venetoclax combination, and grade 3 hyperbilirubinemia and grade 4 diarrhea with the addition of rituximab. The most common grade 3 and 4 side effects were neutropenia (28%), anemia (23%), and thrombocytopenia (23%).

### Potential mechanisms for combination therapy in clinical trials

A potential mechanism for the combination of the BET inhibitor ABBV-075 and the BCL2 inhibitor venetoclax (NCT02391480) in patients with AML is that venetoclax monotherapy increases MCL1 protein levels, whereas combination therapy with ABBV-075 results in a reduction in both MCL1 and Bcl-xL levels and together induces tumor cell apoptosis.^173,174^

The mechanism underlying the synergistic effect of the BET inhibitor AZD5153 with the PARP inhibitor olaparib (NCT03205176) in the treatment of ovarian cancer is that AZD5153 enhances the sensitivity of cells to olaparib and destabilizes genetic material by downregulating PTEN expression, thereby reversing acquired resistance.^175^ Through PAX5 and B-cell antigen receptor mechanisms essential for ABC DLBCL, AZD5153 enhances the efficacy of the Bruton tyrosine kinase inhibitor acalabrutinib in ABC DLBCL cell lines (NCT03527147).^176^

The safety and effectiveness of the combination of ZEN003694 and abemaciclib were evaluated in a Phase I clinical study (NCT05372640), including patients with NUT cancer or other solid tumors. The potential mechanism by which this combination was found to be effective in estrogen receptor (ER)-positive breast cancer was that ZEN003694 treatment reversed the significant overexpression of CDK6 and CCND1 that leads to abemaciclib resistance.^177^

## Adverse events associated with BET inhibitors

While exploring the clinical activity of BET inhibitors, the adverse events related to BET inhibitors require more attention. According to current studies, nausea/vomiting, thrombocytopenia, fatigue, diarrhea, dysgeusia, anemia, decreased appetite, and hyperglycemia are the most common adverse effects of BET inhibitors.^117,120,126,163,165,178–180^ Most of these adverse effects also occur with other anticancer drugs and are tolerated by patients; however, some can have serious consequences and must be treated with caution.

Thrombocytopenia is the most common and serious hematologic adverse effect of dose-limiting toxicity observed clinically with BET inhibitors,^117,120,126,165,178^ and in severe cases, may lead to coagulation failure or even uncontrollable bleeding. This side effect may be related to BET inhibitors interfering with the transcription factor GATA1,^57,181–183^ which is known to have an essential function in maintaining red blood cell function.^184,185^ In clinical trials, BET inhibitor-induced thrombocytopenia was found to be reversed by stopping the therapy for one week after two consecutive weeks of oral OTX015.^117,126^ While it is generally seen that platelet counts tend to recover within a week after discontinuing the medication, this intermittent dosing may reduce drug efficacy and contribute to acquired drug resistance. Another concern associated with increasing BET inhibitor doses to consistently achieve effective plasma drugs concentrations, there may be an increased risk of other dose-limiting toxicities. One approach worth exploring is that combining a BET inhibitor with an antiplatelet-destructive agent may ameliorate this adverse effect.

Studies have demonstrated that BET inhibitor therapy can induce the transactivation of viral genomes, such as the EB virus and Kaposi’s sarcoma-associated herpesvirus.^186–189^ Furthermore, there is a concern that latent human immunodeficiency virus (HIV) could potentially be reactivated as a result of such therapeutic intervention. HIV destroys the individual’s immune system via weakening CD4-positive helper T cells, leaving the host susceptible to pathogenic infections and malignancies.^190^ BRD4 can silence the HIV genome by competing with Tat, a P-TEFb-related viral transactivator, and by inducing CDK9 phosphorylation at threonine 29, which inactivates CDK9.^65,191–193^ BRD2 can silence the HIV genome by introducing the repressor complex E2F1/p50 heterodimer into the HIV promoter or by interacting with the architectural/insulator protein CCCTC-binding factor to form a transcriptional boundary.^194,195^ Treatment with BET inhibitors deregulates the HIV genome by targeting BRD4 and BRD2, leading to the transactivation of HIV. Therefore, it is important to monitor for potential reactivation of viral infections during BET inhibitor treatment.

For male patients using BET inhibitors, testicular atrophy is a concerning adverse effect. As BRDT performs a vital function in spermatogenesis and this role is mainly mediated by BD1, and BRDT and the BD1 structural domain of BRD4 share 81% homology, even modest dosages of pan-BET inhibitors cause testicular atrophy in male mice.^75,196^ This testicular toxicity induced by JQ1 was more pronounced in mature male mice compared to young males, suggesting a possible inhibition of spermatogenesis, an inhibition that appeared to be reversible without affecting hormone levels, a finding that suggests a potential application of BET inhibitors as male contraceptives.^196^ However, whether sperm inhibition by BET inhibitors can be sufficient for contraception, whether testicular function can be fully restored after discontinuation of the drug, and whether fetal malformations can be caused if defective or incompletely restored sperm complete the fertilization process and produce offspring after use of the drug are questions that need to be addressed in further research.

Data from in vivo studies suggest that BRD4 inhibition can also induce a variety of important, albeit reversible, phenotypes, including alopecia, skin hyperplasia, and small intestinal stem cell deficiency.^197^ Furthermore, BET inhibitors also have therapeutic effects on non-tumor cells, raising concerns about collateral damage to healthy tissues from their usage as anticancer drugs. BET inhibitors may affect cardiomyocytes’ BRD4-dependent transcriptional programs, which might help prevent heart failure brought on by hypertrophy of the cardiomyocytes; however, this also highlights the risk of potential adverse cardiac effects.^198–200^

Preclinical data suggest that synergistic therapy may reduce overlapping toxicity issues by enhancing cytotoxicity and enabling every drug to be administered with a lower dosage. As an example, treatment of lymphoma cells with a combination of the BET inhibitor RVX2135 and the histone deacetylase (HDAC) inhibitors vorinostat or panobinostat resulted in apoptosis at doses of each drug that induced only cell cycle arrest when used as monotherapy.^201^ The overall survival of mice that had a transplantation of 2749 lymphoma cells was shown to be significantly enhanced when treated with a combination of RVX2135 and vorinostat, demonstrating a significant synergistic effect.^201^ In addition, vorinostat is effective at lower doses, suggesting that toxicity from the combination may be reduced if the dose of individual drugs can be reduced without compromising the efficacy.^201^ In many cases, the therapeutic activity of BET inhibitors in conjunction with other pharmaceutical agents may be attained at non-cytotoxic dosages, suggesting that using lower doses than when these drugs are administered alone may avoid excessive toxicity in humans. These issues are explored in depth in the subsequent section. Notably, not all combination therapies achieve this considerable effect. For example, when combining BET inhibitors and mTOR inhibitors, thrombocytopenia is a common adverse effect of these two groups of medicines; therefore, the problem of superimposed side effects from both drugs must be considered when using them in combination.^44^

## Resistance to BET inhibitors

Although BET inhibitors have demonstrated efficacy against various cancers, resistance to these inhibitors remains a significant issue in clinical treatment. Tumor or cancer cell insensitivity to BET inhibitors may be due to either primary (or inherent) or acquired resistance resulting from secondary adaptation to treatment. Therefore, exploring the mechanisms underlying resistance to BET inhibitors is essential for optimizing their clinical efficacy.

Several studies have investigated the mechanisms responsible for resistance to BET inhibitors in different cancers. For instance, a 2015 study on leukemia found that in human and mouse leukemia cells, enhanced Wnt/β-catenin signaling partly contributed to BET inhibitor resistance. However, negative regulation of this process led to a return of susceptibility to BET inhibition, as demonstrated both in vitro and in vivo.^202^ In the same year, Rathert et al. also demonstrated that Wnt signaling was a driver and potential biomarker of BET resistance in leukemia, and they suggested that rewiring of the transcriptional program is a key mechanism that promotes resistance to BET inhibitors.^203^ CRISPR-mediated loss-of-function screens have emerged as a robust methodology for the unbiased identification of potential candidates linked to various biological phenotypes.^204–206^ In a combinatorial CRISPR screen of KMT2A rearranged leukemia cell lines, deficiency of the gene encoding *SPOP* was found to cause significant BET inhibitor resistance, which was subsequently proven in a xenograft model.^207^ In a study on TNBC, resistance to BET inhibitors was closely associated with hyperphosphorylation of MED1 and BRD4, which was associated with the decreased activity of protein phosphatase 2A in resistant cells.^208^ BRD4 as well as LSD1/NuRD complexes co-localize at super-enhancers in TNBC, and these complexes limit the over-activation of gene clusters associated with drug-resistance function, including GNA13 and PDPK1.^209^ As a result of the BRD4/LSD1/NuRD complex’s decommissioning due to prolonged treatment or PELI1 instability against LSD1, resistance to JQ1 and other medicinal substances developed.^209^ This suggests that treating TNBC by jointly targeting BRD4 and PELI1 may address the drug-resistance problem to some extent. TAMs are often involved in mediating the resistance of cancerous cells to platinum-based chemotherapy, anti-VEGF/VEGFR treatment, and radiotherapy,^210–213^ and one study discovered that TNBC-stimulated TAMs activate NF-κB signaling by upregulating IKBKE expression to increase breast carcinoma cell resistance to BET inhibitors.^214^ BRD4 hyperphosphorylation, which is associated with CDK9 kinase activity, has also been found in NMC and other cancers. This indicates that BRD4 hyperphosphorylation may be another important mechanism involved in resistance to BET inhibitors.^215^

These findings establish a theoretical foundation for synergizing BET inhibitors with other pharmaceutical agents to enhance their clinical effectiveness. Further investigation is required to have a better understanding of the processes through which medication resistance arises and the steps that work to avoid it. These studies will help develop more effective methods for treating patients who have developed drug resistance and will improve the administration of these medications in combination treatment.

## Combination treatment of BET inhibitors

Although resistance to BET inhibitors is a significant impediment when it comes to clinical use, preclinical studies have shown that resistance to a single-target drug can be overcome when used in combination with other drugs, providing a bright future for BET inhibitors. Based on preclinical data, combining a BET inhibitor with another drug can produce synergistic effects in a variety of ways. There are currently several clinical trials that are investigating the use of BET inhibitors in conjunction with other medications (Table 2). The concurrent administration of BET inhibitors and other pharmaceutical agents also contributes to the mitigation of potential drug toxicity, because of their higher efficacy at lower drug doses.

### Combination with chemotherapy agents

Multiple studies have shown evidence of the synergistic effects of BET inhibitors in combination with cisplatin in ovarian malignancies as well as mouse xenograft models, including those that are resistant to platinum-based treatments and relapsing models.^216–219^ Aldehyde dehydrogenase (ALDH)-positive cells are recognized contributors to tumor progression and relapse following initial chemotherapy response.^220^ This may stem from the differential impact of BET inhibitors, which suppress ALDH activity and ALDH1A1 expression, in contrast to the typical effect of cisplatin.^217^ Furthermore, ovarian cancer cells have exhibited a synergistic response through ALDH1A1 downregulation when combined with platinum-based chemotherapeutic agents.^221^ In addition, it has been demonstrated that BET inhibitors downregulate the expression of Bcl-2 and Survivin, increasing the sensitivity of ovarian cancer cells to cisplatin.^218,222^ Furthermore, the predominant mechanism of action is thought to be DNA damage induction,^223^ and BET inhibitors can decrease the expression and functionality of BRCA1 and RAD51 via inhibiting BRD4 and impair the reporting activity of homologous recombination (HR), thus making HR-proficient ovarian tumor cells more sensitive to DNA damage.^216,224^ Taken together, these findings present a compelling justification for the administration of BET inhibitors when combined with cisplatin for the treatment of HR-proficient ovarian carcinoma.

TNBC is distinguished by the lack of clearly defined molecular targets, and its genomic heterogeneity poses limitations on the available treatment choices for chemotherapy. The use of platinum-based drugs is increasingly being recognized as a viable approach for the development of effective therapeutics targeting TNBC.^225–228^ Studies have shown that BET inhibitors can downregulate BRCA1 and RAD51 expression and impair homologous recombination-mediated DNA damage repair, thereby inducing the functional creation of a BRCAness phenotype in TNBC cells.^224,229^ Findings conducted in vitro have shown that TNBC cells with BRCAness are more sensitive to platinum salts. Clinical trials have also shown enhanced efficacy of carboplatin in TNBC populations with BRCA1/2 mutations.^224,229,230^ Furthermore, it has been shown that BET inhibitors have cytotoxic effects in combination with chemotherapy in in vitro tests on TNBC cells. JQ1 has been shown to have a synergistic interaction with mitotic drugs, such as docetaxel and vinorelbine, as well as with DNA-damaging agents like cisplatin and carboplatin.^230^

In a study on colorectal cancer, the concurrent administration of BET inhibitors together with 5-fluorouracil or oxaliplatin demonstrated a noteworthy enhancement in the effectiveness of treatment in colorectal cancer cell.^231,232^ Recently, the mechanisms underlying these findings have also been explored. Death receptor 5 (DR5) is an essential constituent of the extrinsic apoptotic pathway in colorectal cancer cells.^233^ Studies have shown that depletion of BRD4 and treatment with BET inhibitors significantly induce DR5 expression.^232^ The induction of DR5 is regulated by endoplasmic reticulum stress and CHOP, which is critical for the chemical sensitization and apoptotic effects of BET inhibitors.^232^ Additionally, increased DR5 induction is also responsible for enhancing the sensitivity of colorectal malignancies containing SPOP mutations to BET inhibition.^232^ The BRD4 E3 ubiquitin ligase subunit SPOP is important for regulating this process.^234^ Combining BET inhibitors with chemotherapy effectively inhibited cancer development in a DR5-dependent manner in a colorectal cancer xenograft model; moreover, enhanced DR5 induction and apoptosis could effectively suppress the growth of patient-derived xenograft tumors.^232^

**Fig. 4:**
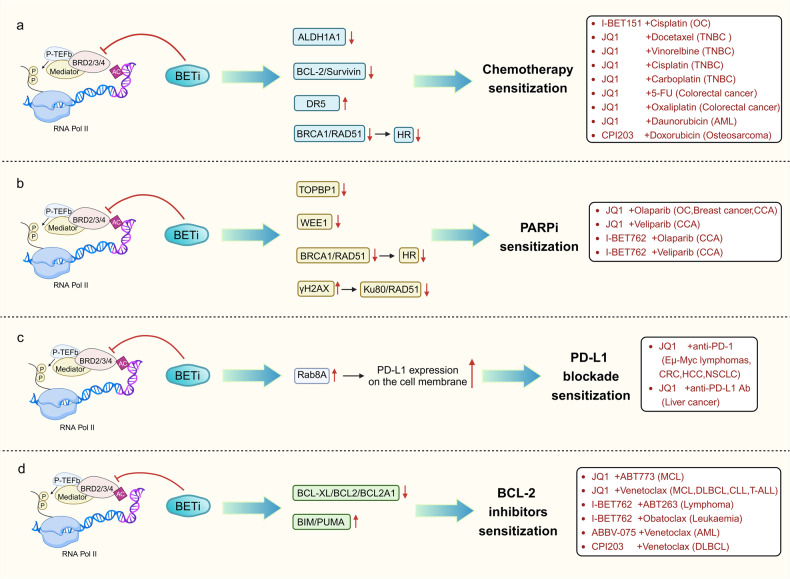
BET inhibitors synergize with a variety of antitumor agents. **a** BET inhibitors in combination with chemotherapeutic agents re-sensitize chemotherapy-resistant tumor cells. The figure summarizes the combinations of BET inhibitors and chemotherapeutic agents that are currently used in multiple tumors. **b** BET inhibitors combined with PARP inhibitors re-sensitize tumor cells resistant to PARP inhibitors. A combination of two drugs currently applied to several tumors is presented in the figure. **c** Inhibition of BET proteins upregulates Rab8A expression, which upregulates PD-L1 expression at the cell membrane, thereby sensitizing PD-L1 blockade. **d** Combined inhibition of BET and BCL-2 re-sensitizes tumor cells to BCL-2 inhibitors. Multiple drug combinations demonstrated synergistic effects in a variety of tumors

In summary, the use of BET inhibitors in conjunction with chemotherapy is a highly effective approach to cancer treatment that can improve outcomes, minimize toxicity, and prevent drug resistance (Fig. 4a). However, it is important to note that this combo treatment may potentially raise the possibility of treatment-related adverse events. Therefore, the selection of the optimal combination therapy regimen requires a comprehensive evaluation of the patient’s individual characteristics, disease status, and tumor type.

### Combination with PARP inhibitors

In preclinical animal models of ovarian carcinoma with HR proficiency, BET inhibitors have demonstrated an ability to heighten tumor sensitivity to PARP inhibitors.^224^ This outcome could potentially stem from BET inhibitors diminishing HR activity while intensifying the DNA damage response prompted by PARP inhibitors. BET inhibitors can impact the transcription of pivotal HR genes like RAD51 and BRCA1.^224^ Additional research has demonstrated that BET and PARP inhibitors exhibit synergistic activity against BRCA1/2 wild-type ovarian malignancies. This can be attributed to the downregulation of DNA topoisomerase II binding protein 1 (TOPBP1) and mitosis inhibitor protein kinase WEE1 by BET inhibitors.^235^ Prior to mitosis, at G2/M cell cycle checkpoints, WEE1 is essential in stopping DNA repair.^236^ Whereas TOPBP1 is crucial for DNA replication and damage signaling.^237^ Downregulation of TOPBP1 renders cells highly sensitive to PARP inhibitors.^238,239^ Hence, directing interventions toward WEE1 and TOPBP1 may make BRCA1/2 carcinoma cells sensitive to PARP inhibitors.

BET inhibitors enhance DNA damage induced by PARP inhibitors.^224^ Investigations conducted in a breast carcinoma model found that the combined use of JQ1 and olaparib markedly inhibited cancer development, which was related to the inhibition of BRCA1 and RAD51 expression by BET inhibitors.^224,229^

As part of a study to better understand their underlying mechanisms, researchers assessed the antiproliferative activity of the PARP inhibitor olaparib and the BET inhibitor JQ1 on PDAC in vitro and in vivo.^240^ The findings indicated that the combined application of JQ1 and olaparib exerted synergistic effects in vitro and were more effective in the independently derived PDX model in vivo than either drug alone.^240^ This could be attributed to JQ1’s ability to decrease the association between BRD4 and BRD2 with Ku80 and RAD51 promoter sites, and data from short hairpin RNA (shRNA) suggested that the expression of Ku80 and RAD51 in PDAC cell lines was dependent on BRD4 and BRD2.^240^ These findings offer a novel strategy of combining BET inhibitors and PARP inhibitors as a promising approach for treating PDAC and may lead to more strong countermeasures against this kind of cancer (Fig. 4b).

Furthermore, researchers evaluated the effects of combining BET inhibitors (JQ1 or I-BET762) with PARP inhibitors (olaparib or veliparib) on the cholangiocarcinoma (CCA) cell lines, as well as the effectiveness of JQ1 and olaparib in a CCA xenograft model.^241^ The results demonstrated that the efficacy of each combination therapy surpassed that of each medicine administered alone.^241^ Mechanistically, downregulation of the BET inhibitor molecular targets BRD2 or BRD4 using shRNA rendered CCA cells sensitive to BET inhibitors as a single drug and in combination with a PARP inhibitor.^241^ These results provide valuable information on the potential of combining BET inhibitors and PARP inhibitors as a novel approach to treating CCA. Moreover, a synergistic effect has been observed between BET inhibitors and PARP inhibitors.^242^

Although the combination of BET inhibitors and PARP inhibitors has shown promising prospects in antitumor therapy, there are also potential risks and limitations. For example, combined use may increase the toxicity of the treatment, which could lead to immune system suppression and liver damage. Therefore, while the combination of BET inhibitors and PARP inhibitors is a promising antitumor treatment approach, further research is needed to validate and refine its effectiveness.

### Combination with immune checkpoint inhibitors

It has been identified that BET inhibitors are suppressors of CD274 (encoding PD-L1), acting at the transcriptional level via the inhibition of BRD4. As a result, there is a downregulation of PD-L1 expression on the surfaces of both tumor cells and immune cells.^243–245^ The combination of BET inhibitors and immune checkpoint inhibitors has demonstrated more effective synergistic effects than monotherapy, both in vivo and in vitro.^244^ Furthermore, evidence supporting the effectiveness of combination treatment was provided by the development of a mathematical model to simulate the combined action of BET and immune checkpoint inhibitors in breast cancer.^246^

BET inhibitors were shown to reduce BRD4 binding to the promoter of the encoded PD-L1 gene, hence downregulating PD-L1 expression levels in a mouse model of Eμ-myc transgenic lymphoma.^244,247,248^ This has led to the idea of combining BET inhibitors with immune checkpoint inhibitors, such as anti-PD-1 antibodies, to target the PD-1/PD-L1 axis. Combining these two types of inhibitors produced a synergistic response in mice harboring MYC-driven lymphomas, indicating an interaction between BET inhibitors and PD-1/PD-L1 immune checkpoints.^244^

Combining JQ1 with anti-PD-L1 antibodies to treat liver cancer in transgenic mice could strongly inhibit tumor progression.^249^ The effectiveness of this combination therapy is associated with BET inhibitor-induced upregulation of Rab8A expression, which can upregulate PD-L1 expression on the cell membrane and sensitize PD-L1 blockade (Fig. 4c).^249^ Overall, this combination therapy promotes the anticancer effect of anti-PD-L1 antibodies and enhances the liver’s anticancer immune response. In a mouse model of primary liver cancer bearing a heart xenograft, researchers explored a combination immunotherapy strategy employing JQ1 alongside the anti-PD-L1 antibody.^250^ The utilization of a combined immunotherapy strategy significantly inhibited the advancement of primary liver cancer without accelerating xenograft rejection.^250^ This presents an innovative and effective treatment strategy for solid organ transplant recipients.

Significant synergistic effects between BET inhibitors and PD-L1 inhibitors have been demonstrated in colorectal cancer,^251^ NSCLC,^252^ and prostate cancer.^253^ The combination of BET inhibitors and immune checkpoint inhibitors is a promising antitumor treatment approach, providing more treatment options for cancer patients. However, combination therapy also has potential risks and limitations. For example, it may increase the toxicity of treatment, leading to immunosuppression and liver damage. Further research is necessary to verify its safety and efficacy and to determine the optimal approach for combination therapy.

### Combination with PI3K inhibitors

Research has shown that BET inhibitors have the ability to enhance the susceptibility of ovarian cancer cells to phosphoinositide 3-kinase (PI3K) inhibitors and inhibit the reactivation of the PI3K pathway subsequent to PI3K inhibitor therapy.^254^ Furthermore, it has been shown that BET inhibitors possess the ability to block receptor tyrosine kinases (RTKs) and the subsequent signaling pathways they activate. However, ovarian cancer cell lines can acquire resistance to BET inhibitors through adaptive reprogramming.^84^ The development of such resistance mechanisms primarily depends on the reactivation of the signaling pathway. Therefore, combination therapy involving BET inhibitors and kinase inhibitors may be a potential strategy for mitigating BET inhibitor resistance and improving the efficacy of BET inhibitor therapy. The synergistic impact of combining ponatinib with BET inhibitors has proven effective in ovarian cancer treatment. The heightened anticancer potency achieved through the combination of ponatinib and BRD4-targeted agents is related to the reduction of MYC expression inside cancer cells.^255^ One compelling study has shown that BET/PI3K combination therapy has synergistic effects in tumor organoid models derived from patients with ovarian clear cell carcinoma, offering a potential treatment option for this rare subtype of chemoresistant ovarian cancer.^256^ Nevertheless, further preclinical research is necessary to verify the effectiveness and security of this approach.

**Fig. 5:**
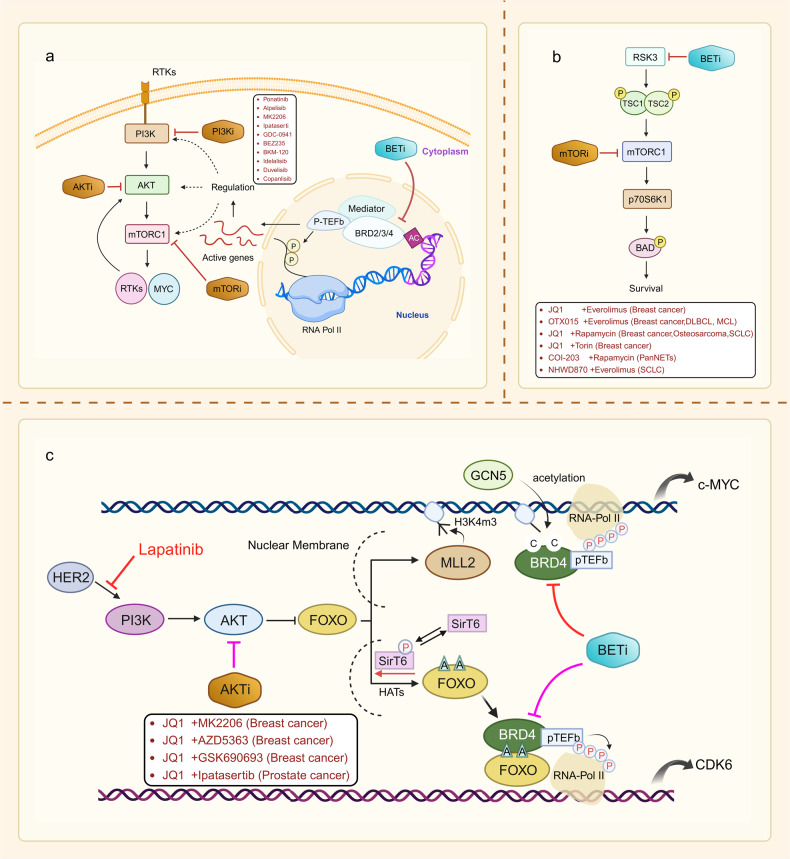
BET inhibitors have synergistic antitumor effects with multiple signaling pathway inhibitors. **a** BET inhibitors block the feedback activation of RTKs, thereby re-sensitizing tumor cells to PI3K inhibitors. The figure shows PI3K inhibitors that have now been used in combination with BET inhibitors. **b** Combined inhibition of mTOR and BET cooperatively promotes tumor cell apoptosis, and synergistic effects of the two drugs have been found for a variety of drug combinations. **c** Mechanism of the synergistic effect of BET inhibitors with lapatinib and AKT inhibitors. In HER2 breast cancer cells, lapatinib inhibits kinases, leading to the activation of FOXO, which in turn translocates to the nucleus and recruits MLL2 and GCN5 to their target genes to add active histone markers, including H3K4m3 and histone acetylation. The modified histones can further recruit BRD4 protein to the target gene, thereby promoting Myc gene transcription to reduce the sensitivity of cancer cells to lapatinib.When cells are treated with AKT inhibitors, FOXO is dephosphorylated and translocates to the nucleus, while dephosphorylated SirT6 loses its ability to bind FOXO, resulting in increased FOXO acetylation. Acetylated FOXO interacts with BRD4 to form a transcriptional activation complex with P-TEFb and RNA polymerase II at the CDK6 gene promoter, activating CDK6 transcription and leading to resistance of breast cancer cells to AKT inhibitors. BET inhibitors target the BRD4 protein to affect Myc and CDK6 transcription, thereby restoring tumor cell sensitivity to lapatinib and AKT inhibitors

Research has indicated that persistent mTORC1 activation diminishes sensitivity to PI3K inhibitors, while inhibiting mTORC1 enhances PI3K inhibitor efficacy and prolongs resistance delays.^257,258^ Resistance to PI3K inhibitors frequently occurs via the mechanism of feedback activation of RTKs, such as the AKT and mTOR pathways (Fig. 5a).^259–263^ RTKs belonging to the epidermal growth factor receptor and insulin receptor families have been identified as direct targets of BRD4 in multiple cancer models.^254^ In these models, the inhibition of BET proteins can effectively regulate the PI3K signaling pathway in breast cancer cells.^254^ This regulation is achieved by preventing the recruitment of BRD4 to the regulatory regions of these receptors, thereby blocking the feedback activation of the RTKs.^254^ The combination inhibition of PI3K/BET in breast cancer cells demonstrated an effectively curbed reactivation of AKT and mTORC1, re-sensitizing the cells to PI3K inhibitors.^254^ This combination therapy’s effectiveness was also substantiated through an in vivo mice model of breast cancer.^254^ Researchers studying breast cancer cells discovered that combining therapy with JQ1 and the dual PI3K/mTOR inhibitor BEZ235 could prevent chromatin structure alterations, prevent malignancy cells from developing resistance, and cause xenograft regression in vivo as well as in vitro cell death.^264^ Significantly, this combination also exhibited remarkable synergy in hepatocellular carcinoma^265^ and colorectal cancer,^266^ showcasing the effectiveness of BET inhibitors in countering tumor resistance to dual PI3K/mTOR inhibition. This discovery offers an innovative and effective treatment strategy for cancer therapy. Notably, this combination treatment can also inhibit the development of drug-tolerant persisters, making therapy more durable and effective.^264^

Activation of PI3K performs an essential function in the pathogenesis of lymphomas driven by the MYC oncogene, making it a prospective target for therapeutic intervention.^267–269^ Several studies have investigated the effectiveness of combined treatment, including BET and PI3K inhibitors, in various lymphomas, such as DLBCL^97,101,270^ Burkitt’s lymphoma (BL),^270,271^ MCL,^272,273^ marginal zone lymphoma,^273^ chronic lymphocytic leukemia (CLL),^273,274^ peripheral T-cell lymphoma,^273^ and mouse MYC-stimulated lymphoma cells.^275^ Combination therapies involve multiple mechanisms of action. First, BET inhibitors can improve the antiproliferative effect of PI3K inhibitors by increasing GSK3β S9 phosphorylation levels and β-catenin abundance through the downregulation of the E2 ubiquitin-conjugating enzymes UBE2C and UBE2T.^270^ Second, PI3K inhibitors upregulate various constituents within the B-cell antigen receptor pathway, whereas BET inhibitors downregulate some of these components (e.g., SYK, BLK, CD79A, and CD79B), thereby counteracting the adaptive response induced by idelalisib.^276,277^ Additionally, NF-κB activity is critical for the viability of some malignant cells and is maintained by the constitutive activity of IKK in the cytoplasm.^278^ By suppressing the expression of BRD2 and BRD4, BET inhibitors reduce IKK activity and block the downstream NF-κB-driven transcriptional program, which may strengthen the combined effect.^97,101^

### Combination with mTOR signaling pathway inhibitors

Everolimus is an effective and safe oral inhibitor of mTOR, utilized in the treatment of breast carcinoma patients.^279^ This occurrence of resistance to everolimus has been linked to MYC upregulation, which is associated with higher BRD4 recruitment to MYC in everolimus-resistant cell lines.^280,281^ BET inhibition has been found to restore sensitivity in cells that are resistant to mTOR inhibitors.^281^ in vitro and in vivo studies have revealed that the combined therapy of everolimus and JQ1 exhibits higher effectiveness compared to the use of either drug alone.^281^ in vitro and xenograft models exhibited that OTX015, an innovative BET inhibitor, had synergistic antiproliferative effects when combined with everolimus.^282^ Additionally, the mTOR inhibitors rapamycin and Torin were previously found to enhance the susceptibility of JQ1-resistant cells to BET inhibition.^280^

mTOR has become a crucial therapeutic target in certain hematolymphoid tumors.^283^ BET inhibitors, when combined with mTOR inhibitors, have also shown strong synergistic effects against a variety of lymphomas and solid tumors.^44,97,101,271,275,276,284,285^ One possible mechanism is the inhibition of BET proteins, which leads to the restoration of sensitivity in drug-resistant cells towards mTOR inhibitors. However, since thrombocytopenia is a common toxicity of these two types of drugs, it is necessary to pay attention to drug side effects when using them in combination.^44^

A study using various molecular subtypes of patient-derived SCLC xenograft models demonstrated that, without appreciably raising toxicity, mTOR inhibition enhances BET inhibition’s anticancer activity in vivo.^286^ Moreover, BET inhibition induces apoptosis in SCLC models both in vitro and in vivo; the anticancer effect is further enhanced by cotreatment with mTOR inhibition.^286^ Activation of the intrinsic apoptotic pathway is the mechanism through which BET inhibition induces apoptosis in SCLC cells.^287^ BET inhibition upregulates RSK3, which activates the TSC2/mTOR/p70S6K1/BAD cascade to promote cell survival; however, this protective signaling can be blocked by mTOR inhibition to enhance BET inhibition-induced apoptosis (Fig. 5b).^286^

### Combination with MEK inhibitors

Research has demonstrated evidence for synergistic benefits when treating ovarian cancer in vivo and in vitro with BET and MEK inhibitors, such as JQ1 and PD0325901 or trametinib, combined.^288^ BET inhibitors have been shown to induce cell cycle arrest rather than apoptosis in ovarian cancer cells, which may impose limitations on the potential therapeutic application for individuals with advanced ovarian cancer.^288^ However, the combined use of BET and MEK inhibitors can circumvent this constraint since co-administration of MEK inhibitors can nullify the BET inhibitor-induced feedback activation of the MAPK signaling pathway.^288^ Research has shown that the co-administration of BET and MEK inhibitors can coordinate the regulation of apoptotic molecules, such as BIM and BAD, and induce tumor cell apoptosis in ovarian cancer cells.^288^ Moreover, combining BET inhibitors with MEK inhibitors can also overcome resistance resulting from kinome reprogramming in neurofibromatosis type 1 (NF1)-deficient ovarian cancer cells, where MEK inhibitors transcriptionally induce the reactivation of RTK and its downstream pathways.^289^ Large-scale genomic studies have revealed that NF1, a RAS guanosine triphosphatase (GTPase)-activating protein that negatively regulates RAS family proteins, is lost in 12% of epithelial ovarian tumors.^290,291^ The loss of NF1 leads to the reactivation of RAS effector pathways, including PI3K/mTOR/AKT, RAF/MEK/ERK, and RALGDS signaling, which contributes to neoplastic proliferation and survival.^290,292,293^ MEK inhibitors trigger the transcriptional reactivation of RTK and its downstream pathways by destabilizing FOSL1, encompassing the RAF/MEK/ERK, JAK/STAT, and PI3K/AKT signaling pathways.^289^ However, in epithelial ovarian cancer cells lacking NF1, MEK inhibitor-induced RTK reprogramming renders them resistant to trametinib treatment, thereby reducing the growth inhibition of tumor cells by MEK inhibitors.^289^ BRD2 and BRD4 have been discovered to be pivotal players in the transcriptional regulation of RTKs in NF1-deficient cells, and functional inhibition of the BET protein has demonstrated the ability to counteract MEK inhibitor-triggered elevation of RTK RNA and protein expression.^289^ Therefore, BET and MEK inhibitors combined might serve as a beneficial therapeutic approach for NF1-deficient ovarian carcinomas; however, it is crucial to recognize that this combination therapy may not be effective for epithelial ovarian cancer cells that are NF1-sufficient.^289^ Overall, the combined use of BET and MEK inhibitors suggests a potentially advantageous strategy for addressing ovarian cancer therapeutically.

In TNBC, trametinib inhibits MEK to block the MEK/ERK pathway, resulting in a robust initial growth suppression. However, suppression of MEK/ERK triggers the activation of an alternate kinase pathway that inhibits growth. This upregulation allows the growth arrest to be overcome by an adaptive bypass response.^294^ BET inhibitors make trametinib-resistant cells more sensitive to the drug by inhibiting BRD4 and effectively reverse trametinib resistance by inhibiting the adaptive upregulation of RTKs.^295^ BET inhibitor and trametinib combined therapy produced synergistic growth inhibitory effects in different TNBC mouse models in vitro and in vivo.^295^ The concurrent administration of BET and MEK inhibitors has a synergistic effect on reducing the viability of TNBC cell lines and patient-derived xenograft models, suggesting that this treatment strategy may be a promising therapeutic option.^296^ Of particular note, this study found that cells exhibiting elevated expression levels of MYCN demonstrate heightened sensitivity towards BET inhibitors,^296,297^ providing a basis for extending the use of BET and MEK inhibitors in individuals who suffer from late-stage TNBC expressing MYCN.^296^ The finding has the potential to open up novel avenues for the development of more precise therapeutic interventions.

Limited research supporting the synergistic antitumor effects of BET and MEK inhibition in melanoma exists.^298,299^ One possible mechanism is that the co-administration of BET and MEK inhibitors can alleviate the transcriptional characteristics of MAPK and checkpoint inhibitor resistance, downregulate the transcription factor TCF19, and induce cell apoptosis.^298^

### Combination with tyrosine kinase inhibitors

Lapatinib is a small-molecule tyrosine kinase inhibitor that targets HER2 as well as the epidermal growth factor receptor.^300,301^ It was approved by the FDA in 2007 for the treatment of advanced or metastatic breast cancer that overexpresses HER2 and was previously treated with anthracyclines, taxanes, and trastuzumab.^300^ However, resistance to lapatinib significantly limits its effectiveness against breast cancer.^302^ FOXOs are tumor suppressors that upregulate the expression of cyclin-dependent kinase inhibitors and pro-apoptotic proteins.^303,304^ When the PI3K/AKT signaling pathway is activated, the protein kinase AKT directly phosphorylates FOXO at three conserved sites, leading to the translocation of FOXO from the nucleus and inhibition of transcriptional activity.^305^ Lapatinib treatment upregulates MYC by inhibiting AKT-mediated phosphorylation of FOXO, and FOXO-mediated overexpression of MYC reduces the susceptibility of breast carcinoma cells to lapatinib.^306^ The sensitivity of HER2-positive cell lines and HER2-positive xenograft models to lapatinib can be enhanced by BET inhibitors via the reduction of lapatinib-induced upregulation of MYC (Fig. 5c).^306^ Another adaptive response contributing to lapatinib resistance involves kinome reprogramming through the reactivation of ERBB2/ERBB3 signaling, transcriptional upregulation, and activation of multiple tyrosine kinases.^307^ BET inhibitors can re-sensitize breast cancer cells to lapatinib by dissociating BRD4 and RNA Pol II from lapatinib-induced kinase genes.^307^ In summary, the use of a combined therapeutic strategy with BET inhibitors and lapatinib has promise as a more effective therapy modality for individuals diagnosed with breast carcinoma.

**Fig. 6:**
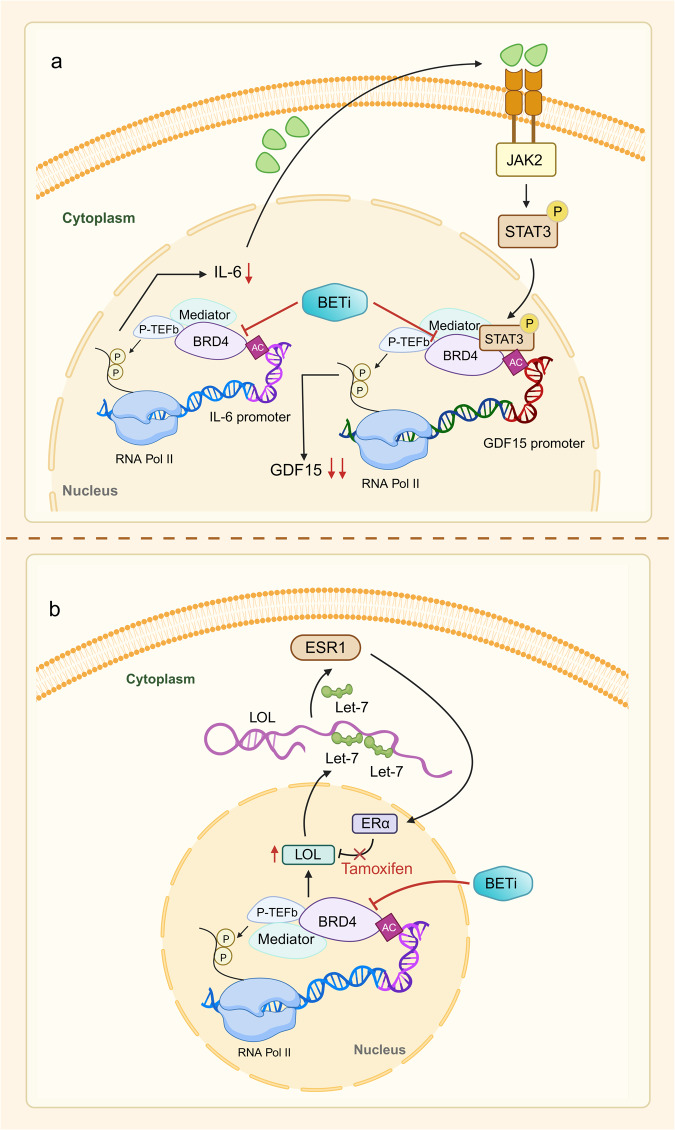
Mechanistic modeling of the synergistic effect of BET inhibitors with sunitinib or tamoxifen. **a** BET inhibitors sensitize melanoma cells to Sunitinib by inhibiting GDF15 expression, either directly through inhibition of BRD4 transcription or indirectly through the BRD4/IL-6/STAT3 axis. **b** BET inhibition re-sensitizes ER-positive breast cancer cells to tamoxifen by reducing LOL expression

Sunitinib serves as an innovative oral multi-targeted tyrosine kinase inhibitor that received approval from the FDA in 2006 for the treatment of individuals with clear cell renal cell carcinoma or gastrointestinal stromal tumors.^308^ Prior research has demonstrated the therapeutic effect of sunitinib on melanoma, which is further enhanced by propranolol.^309^ BET inhibitors can hinder the development of melanoma through the noncanonical NF-κB/SPP1 pathway.^103^ Research has shown that the co-administration of BET inhibitors with sunitinib elicits a synergistic inhibitory effect on melanoma in vitro and in vivo.^310^ The main mechanism is the sensitization of melanoma cells to sunitinib via the inhibition of BET proteins.^310^ This inhibition leads to the suppression of GDF15 expression, which is controlled either directly through BRD4 transcription or indirectly through the BRD4/IL-6/STAT3 axis (Fig. 6a).^310^ This provides a promising alternative treatment option for melanoma.

### Combination with histone deacetylase (HDAC) inhibitors

HDACs are of paramount importance in the context of malignancy because they deacetylate histones and non-histone proteins that regulate the cell cycle, apoptosis, DNA damage response, metastasis, angiogenesis, and autophagy.^311^ HDAC inhibitors can counteract the abnormal acetylation state in cancer cells and activate tumor suppressors.^311^ In comparison to therapy with JQ1, mocetinostat, or valproic acid alone, the combined use of HDAC and BET inhibitors demonstrated synergistic benefits in breast cancer cell lines and significantly reduced cell viability.^312^ It was discovered that TNBC cells were more sensitive to mocetinostat-JQ1 and valproic acid-JQ1 combinations than to ER-positive cells.^312^ The main mechanism underlying this combination treatment is the attenuation of the RAS/MAPK signaling pathway and the upregulation of ubiquitin-specific peptidase 17, leading to decreased cell viability.^312^

HDAC and BET inhibitors have been demonstrated to elicit growth arrest and apoptosis in DLBCL,^97,285,313^ MCL,^272^ and cutaneous T-cell lymphoma^314^ cell lines with good synergistic activity. One possible mechanism for this combination is the application of HDAC inhibitors in AML, which results in histone hyperacetylation.^315^ Simultaneous histone hyperacetylation increases cellular dependence on BET proteins, rendering them sensitive to BET inhibition.^316^ Additionally, BET and HDAC inhibitors combined significantly attenuated the expression of MYC and *BCL2*, thereby increasing apoptosis induction.^314^ Novel compounds that possess the ability to simultaneously target BRD4 and HDAC proteins have been synthesized, taking advantage of the synergistic effects seen when combining BET and HDAC inhibitors.^317–320^

### Combination with BCL2 inhibitors

BET inhibitors and BCL2 inhibitors showed significant synergy in several cancer cell lines, including DLBCL,^173,313,321^ MCL,^272^ CLL,^322^ cutaneous T-cell lymphoma, acute leukemia,^323^ and multiple myeloma.^173^ Treatment with both drugs induces apoptosis more than treatment with either drug alone, and BET inhibitors increase cancer cell sensitivity to BCL2 inhibitors.^173,313,314,321,323^ These effects may occur because of BET inhibitors that enable the downregulation of different anti-apoptotic proteins, including BCL-XL, BCL2, and BCL2A1, and the upregulation of pro-apoptotic proteins like BIM and/or PUMA, in leukemia, lymphoma, and multiple myeloma cell lines (Fig. 4d).^173,313,314,321,323^ In a study of germinal center B-cell (GCB)-like DLBCL cell lines, researchers found an inverse relationship between sensitivity to the BET inhibitor ABBV-075 and sensitivity to the BCL2 inhibitor venetoclax.^173^ Further, the level of apoptosis induced by BET inhibitor treatment of GCB-DLBCL cells was negatively correlated with BCL2 expression.^173^ These findings suggest that BET and BCL2 inhibitors might exhibit complementary effects in the GCB-DLBCL individuals and that BCL2 expression levels or previous responses to BCL2 inhibitors can be used to identify GCB-DLBCL subpopulations that are more likely to undergo apoptosis when treated with BET inhibitors.^173,324^

### Combination with ATR inhibitors

ATR inhibitors lead to enhanced DNA damage and the initiation of apoptosis, whereas BET inhibitors induce DNA damage as well as the upregulation of genes involved in DNA repair.^275,276^ In studies on mouse MYC-induced lymphoma BL^275^ and MCL^276^ cell lines, it was found that the synergistic effects of BET and ATR inhibitors combined. The primary mechanism is that combined treatment of B-cell lymphomas with ATR and BET inhibitors results in gene expression profiles similar to those of the senescence-associated secretory pathway and endoplasmic reticulum stress.^275^ BET inhibitors enhance ATR inhibitor-induced cellular stress and cell death.^275^ Two main ATR inhibitors are currently being investigated in clinical trials,^325,326^ and although some toxicities (e.g., thrombocytopenia and anemia) may overlap, the combination of BET and ATR inhibitors holds immense potential.

One study on melanoma found that both human and mouse melanoma cells were sensitive to combination therapy with ATR and BET inhibitors in vitro and in vivo, possibly due to their synergistic induction of cell apoptosis and senescence-associated secretory pathway.^327^ This provides novel insights and directions for the treatment of melanoma and may serve as a reference for the treatment of many additional tumor forms.

### Combination with AKT inhibitors

AKT inhibitors induce the expression of CDK6 by binding to the forkhead box class O 3a (FOXO3a)/BRD4 combination located at the CDK6 promoter.^328^ CDK6 is an oncogenic kinase that controls G1/S phase transition and cell cycle progression.^329,330^ Cell lines that express high levels of CDK6 exhibit increased RB phosphorylation and disruption of cell cycle arrest, rendering them resistant to AKT inhibitors.^328^ Resistance to PI3K/AKT inhibition can be promoted by the FOXO3a/BRD4/CDK6 axis. However, the combined inhibition of AKT and BET, which blocks the simultaneous PI3K/AKT and FOXO3a/BRD4/CDK6 pathways, proves to be more efficacious compared to monotherapy (Fig. 5c).^328^ in vivo experiments in mice have also confirmed that BET inhibitors sensitize luminal breast cancer cells to AKT inhibitors.^328^ In breast cancer cell lines, on the other hand, overexpression of AKT3 is a common characteristic of acquired resistance to AKT inhibitors, and BET inhibitors can reverse this effect by inhibiting AKT3 expression.^331^

Mutations in the gene encoding the substrate-binding adapter protein POZ of the E3 ubiquitin ligase SPOP are the most common in primary prostate cancer.^332^ Prostate cancer-specific SPOP mutations impair BET degradation, making cells more resistant to cell growth inhibition and apoptosis induced by BET inhibitors.^145,234^ Research has shown that BRD4 accumulation triggers an increase in the expression of GTPase RAC1 and genes involved in the cholesterol biosynthetic pathway, leading to the activation of the AKT/mTOR complex 1 (mTORC1) signaling pathway and the development of resistance to BET inhibitors.^333^ However, resistance can be overcome by the synergistic use of BET inhibitors in conjunction with AKT inhibitors, and this strategy has been shown to be effective against SPOP-mutant prostate cancer.^333^

### Combination with BTK inhibitors

Inhibition of BTK is a well-established treatment for B-cell malignancies. BET inhibitors exhibit strong synergy with ibrutinib in B-cell lymphomas.^276^ Such effects have been noted across a range of lymphoma types, including DLBCL,^101,284,334–337^ MCL,^272,276,336,337^ BL,^271^ CLL, and anaplastic large T-cell lymphoma models.^274^ One plausible explanation for this synergy lies in the on-target side effects associated with BET inhibitors, which may restrict the obtainable clinical dosages. Combining BET inhibitors with ibrutinib could enable achieving comparable antitumor responses at lower doses, thereby mitigating overall toxicity. Another possible explanation is that although ibrutinib showed effectiveness in B-cell lymphoma, most patients didn’t observe a sustained response. Therefore, the efficacy of ibrutinib could be improved by a combination of BET inhibitors. In anaplastic large T-cell lymphoma, combining ibrutinib with a BET inhibitor yielded enhanced interleukin-2-inducible T-cell kinase (ITK) downregulation, leading to a more potent ITK inhibition.^334^

### Combination with endocrine therapy

Compared with parental cells, BET inhibitors selectively inhibit the expression of ER alpha by inhibiting BRD3/4 and attenuate the growth of tamoxifen-resistant, ER-positive cells to an even larger extent.^89^ In addition, transcriptome array analysis of paired luminal breast cancer tumors showed that ENST00000456526, a luminal long non-coding RNA (lncRNA), is overexpressed in luminal breast cancer.^338^ ER-positive tamoxifen-resistant cells exhibit a higher level of lncRNA of luminal (LOL) expression compared with parental cells, and downregulation of LOL enhances the sensitivity to tamoxifen treatment in tamoxifen-resistant breast cancer cells and xenograft models (Fig. 6b).^338^ BET inhibition reverses tamoxifen resistance in ER-positive cells by reducing LOL expression.^338^ In conclusion, BET inhibitor-induced LOL knockdown may represent a potentially effective strategy for hindering the progression of luminal breast cancer and overcoming tamoxifen resistance in patients exhibiting a high level of LOL expression. Furthermore, JQ1 and fulvestrant combined hindered the proliferation of tumors in a tamoxifen-resistant murine xenograft model.^89^

ESE-15-ol is an antimitotic compound that interferes with microtubule dynamics in cells during active division and induces mitotic cell cycle arrest followed by apoptosis.^339^ The combined use of BET inhibitors and ESE-15-ol exhibited considerable synergistic activity against TNBC cells.^340^

### Combination with Aurora kinase inhibitors

High-throughput drug screening has revealed that the combination of BET and Aurora kinase inhibitors is potentially effective in the treatment of ovarian cancer.^288^ The combination of the BET inhibitor JQ1 and Aurora kinase inhibitors has shown promising synergistic effects in drug-resistant ovarian cancer cell lines.^341^ However, further investigation is necessary to elucidate the underlying mechanisms, and clinical trials are warranted to assess the efficacy of this combined therapy for ovarian cancer treatment.

### Combination with EZH2 inhibitors

A study on aggressive B-cell lymphomas identified a feed-forward loop involving MYC, miRNA, and EZH2; MYC overexpression facilitated the recruitment of EZH2 to the miR-26a promoter, resulting in a synergistic suppression of miR-26a.^342,343^ BET and EZH2 inhibitors were found to disrupt MYC activation, leading to synergistic inhibition of lymphoma proliferation and clonogenicity in aggressive lymphoma cells.^342^ Another study found that BET inhibitors downregulated EZH2 and H3K27me3, a marker of EZH2 activity, in DLBCL cells. Stronger downregulation was observed when BET and EZH2 inhibitors were combined.^284^ This combination therapy may be promising for addressing therapies aggressive B-cell lymphoma.

Aberrant activity of EZH2 and BRD4, two H3K27 modifiers, is a key driving factor in atypical teratoid/rhabdoid tumors (AT/RT), and targeted inhibition of these modifiers represents a potential therapy approach for AT/RT treatment.^344–349^ In AT/RT cells, H3K27me3 and H3K27ac levels are elevated and distributed at different chromatin regions to regulate specific gene expression and promote tumor growth.^346,350^ The proliferation of AT/RT cells is suppressed, their invasiveness is reduced, and the levels of H3K27me3 and H3K27ac are decreased by the specific suppression of EZH2 and BRD4 activity.^351^ The concurrent inhibition of EZH2 and BRD4 shows superior in vitro and in vivo therapeutic benefits, surpassing the therapeutic outcomes seen with individual agent treatments.^351^

The combination of BET and EZH2 inhibitors demonstrated superior tumor growth inhibition in diffuse intrinsic pontine glioma via blocking proliferation and promoting apoptosis in vitro as well as in vivo.^352^

A recent study assessed the combination of EZH2 and BET inhibitors within metastatic prostate cancer cells and found superior reduced cell viability, proliferation, and clonogenicity compared with single-agent treatment, while c-MYC and NF-kB expression were reduced.^353^ While this finding provides a novel approach to treating metastatic prostate cancer, the efficacy and safety of this combination require further validation via in vivo experiments and preclinical studies.

### Combination with CREBBP/EP300 inhibitors

BET and CREBBP/EP300 inhibitors combined also demonstrated significant synergistic activity and increased cell death rates.^354^ A recent study on multiple myeloma cell lines found that the novel dual BET and CREBBP/EP300 inhibitors, NEO2734 and NEO1132, exhibited stronger in vitro antitumor effects compared with individual inhibitors targeting either BET or CREBBP/EP300.^355^ In multiple myeloma cell lines, the dual inhibitors induce significant G1 cell cycle arrest and reduce c-MYC and IRF4 protein levels, indicating their potential as a therapeutic option for multiple myeloma.^355^ NEO2734, a novel antitumor compound, has demonstrated superior antitumor activity in lymphoma, leukemia, and prostate cancer.^356,357^

The newly developed inhibitor XP-524, which targets EP300/CBP of histone acetyltransferase and BRD4, demonstrated higher potency and superior antitumor activity compared with single-agent BET inhibition in PDAC.^358^ In addition to inhibiting KRAS/MAPK signaling, XP-524 enhances self-peptide presentation and tumor recruitment of cytotoxic T lymphocytes, despite their refractory nature, even after full activation.^358^ Notably, one study found that the combination of XP-524 with an anti-PD-1 antibody in vivo reactivated the cytotoxic immune program and significantly prolonged survival.^358^ This suggests that XP-524 has promise as a novel treatment alternative for PDAC, and its combination with immune checkpoint inhibitors warrants further investigation.

### Combination with PRMT5 inhibitors

PRMT5 overexpression has been regularly documented in cell lines for lymphoma and leukemia, and its increased expression has been shown to induce the symmetrical methylation of H3R8 and H4R3. Furthermore, studies on MCL have found that PRMT5 overexpression correlates with decreased miR-92b and miR-96 expression.^359^ Research has shown that selective inhibition of PRMT5 expression produces anti-lymphoma activity.^360^ BRD4 binds to the 5’ regulatory region of PRMT5, and treatment with BET inhibitors mis localizes BRD4 from this region. This downregulates arginine methyltransferase expression and reduces PRMT5 occupancy at the cancer suppressor miR-96-5p promoter, leading to the inhibition of miR-96-5P.^361^ This suggests that combining BET and PRMT5 inhibitors could be a new strategy for lymphoma treatment.

### Combination with PLK inhibitors

In a study on BET inhibitor-acquired resistance in TNBC cell models, BET inhibitor-resistant cells were found to be particularly sensitive to PLK1 inhibition.^362^ Inhibition of PLK1 exhibited an antiproliferative effect in resistant cells, causing G2/M arrest and upregulation of the cell cycle proteins Bcl-2, pH3, and cyclin B, along with the induction of caspase-dependent apoptosis.^362^ This suggests that BET and PLK1 inhibitors combined may provide a new treatment option for BET inhibitor-resistant patients.

BET proteins are central regulators of AR and MYC-mediated transcription in CRPC,^88^ whereas PLK1 inhibitors not only affect the cell cycle but can also downregulate AR and MYC.^363,364^ Hence, a novel dual inhibitor of BET and PLK1, WNY0824, was synthesized, exhibiting nanomolar-level and equipotent inhibition against both BRD4 and PLK1.^365^ WNY0824 has demonstrated superior antitumor activity both in vitro and in vivo, providing an innovative therapeutic option for the treatment of CRPC.^365^

## Conclusion

BRDs constitute a dynamically evolving class of epigenetic targets, with aberrant BET protein activation strongly linked to various diseases, including cancer. The emergence of BET inhibitors has yielded significant insights into the pivotal role of BET proteins in governing proto-oncogene transcription, underscoring their therapeutic potential. In recent decades, dozens of clinical trials have been conducted to validate the potential advantages of BET inhibitors in refractory cancer and non-cancerous ailments, yielding initial efficacy. However, addressing drug resistance and adverse effects remains a priority for future drug development. Rational drug combinations have emerged as a promising strategy against drug resistance, validated through clinical trials. Thrombocytopenia stands as a principal safety concern in ongoing clinical trials, and optimizing dosing schedules could ameliorate patient tolerability. The development of BD1- or BD2-selective inhibitors might enhance tolerability, and the advent of BET PROTACs could potentially offer enduring antitumor efficacy.

Preliminary evidence from preclinical and clinical studies suggests that BET inhibitors do not provide durable cytotoxic effects in human cancers when used as single agents. However, the therapeutic potential associated with combining targeted small-molecule inhibitors, immune checkpoint inhibitors, and other epigenetic therapies is significant (Fig. 5). While the potential toxicity of BET inhibitors could restrict this combination, research indicates that employing combination therapies at lower doses might offer effectiveness while mitigating toxicity concerns. In conclusion, BET inhibitors hold significant clinical promise in combination regimens for the future. Enhanced binding approaches and the evolution of next-generation compounds, extending beyond bromodomain targeting, are poised to unlock fresh avenues for the prospective clinical advancement of BET inhibitors as anticancer agents.

An additional critical concern pertains to the absence of biomarkers for predicting BETi sensitivity and the utilization of non-clinically applicable dosages in preclinical investigations, thereby constraining the translation of these agents into clinical practice. Several new approaches are now available to aid in the screening of BET inhibitors for personalized therapy and combination regimens that may improve clinical outcomes.^366,367^ Further mechanistic studies can help identify these biomarkers, and the development of novel, highly selective BET inhibitors will help prevent toxicity.
